# Inflammatory Bridges: Interconnections Between Alzheimer’s Disease and Bone Health

**DOI:** 10.14336/AD.2025.10319

**Published:** 2025-03-19

**Authors:** Hengrui Li, Wendi Wang, Liping Zhang, Tianwei Wang, Jian Liu, Jingguo Wu, Qingbin Ni, Baoliang Sun, Jingyi Sun

**Affiliations:** ^1^Shandong First Medical University & Shandong Academy of Medical Sciences, Taian, Shandong, 271016, China.; ^2^The Second Affiliated Hospital, Shandong First Medical University & Shandong Academy of Medical Sciences, Taian, Shandong, 271000, China.; ^3^Institute of Brain Science and Brain-Inspired Research, Shandong First Medical University & Shandong Academy of Medical Sciences, Jinan, Shandong, 250117, China.; ^4^Department of Neurology, Shandong Second Provincial General Hospital, Jinan, Shandong, 250022, China; ^5^The Affiliated Taian City Central Hospital of Qingdao University, Taian, Shandong, 271000, China.; ^6^Shandong Provincial Hospital Affiliated to Shandong First Medical University, Jinan, Shandong, 250021, China

**Keywords:** Alzheimer's Disease, Bone Diseases, Neuroinflammation

## Abstract

Alzheimer's disease is a neurodegenerative disorder that predominantly affects the elderly and is characterized by complex pathogenesis. Within the unified framework of the ANT, the introduction of B (the disruption of the blood-brain barrier) facilitates communication between the brain and the periphery through the blood-brain barrier. In this context, inflammatory factors act as a bridge, and the neuroinflammation hypothesis is gaining increasing acceptance. This hypothesis involves the abnormal activation of microglia, the release of inflammatory mediators, and damage to astrocytes, which leads to blood-brain barrier impairment and subsequently triggers systemic inflammation. Bone health is associated with conditions such as osteoporosis, osteoarthritis, rheumatoid arthritis, and periodontitis. This hypothesis establishes a link between Alzheimer's disease and bone health. The present article aims to construct an inflammatory bridge between brain and bone health by summarizing the shared mechanisms between the two, specifically focusing on age-related NLRP3-induced pyroptosis, advanced glycation end products, oxidative stress, macrophage autophagy and lysosomal function, and calcium ion dysregulation. Additionally, it reviews the latest therapeutic approaches to explore potential clinical treatments related to the connection between Alzheimer's disease and bone health through these shared mechanisms.

## 1. Introduction

Alzheimer's Disease (AD) is a common and irreversible disorder of the central nervous system, typically affecting the elderly population. The primary symptoms in AD patients include memory loss, cognitive impairment, difficulties in language expression, behavioral changes, and ultimately, the loss of the ability to perform daily living activities independently [1, 2].

Atypical AD encompasses various subtypes, whose clinical manifestations and neuropathological characteristics differ from those of typical AD [1]. Common types of atypical AD include posterior cortical atrophy (PCA), primarily characterized by difficulties in visuospatial abilities and object recognition, with relatively preserved early memory functions; primary progressive aphasia (PPA), which involves a gradual loss of language abilities and is further classified into three subtypes: nonfluent/agrammatic (nfvPPA), semantic (svPPA), and logopenic (lvPPA); behavioral variant Alzheimer's disease (bvAD), marked by changes in mood and behavior, such as apathy, depression, and impulsive behaviors, while memory functions may remain intact; executive dysfunction variant (dexAD), primarily exhibiting a decline in executive functions while preserving memory and visuospatial abilities; and corticobasal syndrome (CBS), often accompanied by motor impairments such as rigidity and bradykinesia, along with potential visual, language, and executive function deficits. Cognitive impairments in AD patients may increase the risk of falls, potentially leading to severe consequences such as fractures [3].

With the global population aging rapidly, the prevalence of Alzheimer's Disease continues to rise, imposing significant medical and economic burdens on individuals, families, and society [4]. Currently, approximately 55 million people worldwide are affected by Alzheimer's Disease, and this number doubles every five years. By 2050, the number of cases is projected to reach approximately 100 million [5]. Although the exact etiology of Alzheimer's Disease is not yet fully understood, research indicates that its pathogenesis is associated with multiple factors, among which the most critical include the deposition of β-amyloid protein (Aβ) [6,7], abnormal phosphorylation of Tau protein [8], neurodegenerative lesions [9], and disruption of the blood-brain barrier [10]. These pathological features can trigger neuroinflammation [11, 12], and increasing evidence suggests that various cytokines, including IL-6, IL-1β, TNF-α, TGF-β, and IFN-γ, actively participate in AD pathogenesis and may serve as diagnostic or therapeutic targets for AD neurodegeneration [12-14]. Genetic factors, including pathogenic genes such as PSEN1, PSEN2, APP, and Sorl1, contribute to AD [15]. Risk genes include one or two alleles of APOE4, and GWAS studies have identified 40 other risk genes [16]. Protective genes include APOE2 [17], as well as PLCG2, KLOTHO [18, 19], and the Icelandic APP A673T gene mutation [20, 21]. Environmental factors [22] and lifestyle choices [23] also significantly contribute to AD development.

With growing research on the human skeletal system, bones are increasingly recognized as endocrine organs [11]. Beyond providing structural support, bones influence metabolism, the immune system, and the nervous system through the secretion of various bone-derived factors [24], such as hormones secreted by osteoblasts and osteoclasts [25]. Increasing evidence suggests that skeletal health is closely associated with the onset and progression of AD [26]. Conditions such as reduced bone density and osteoporosis are linked to cognitive dysfunction, and bone-derived factors may influence AD progression by regulating neuro-inflammation, neuronal survival, and cerebrovascular health. Therefore, investigating the relationship between the skeletal system and the inflammatory bridge in AD may help elucidate potential pathological mechanisms underlying this complex disorder. Additionally, lifestyle factors such as diet, exercise, and cognitive training play a role in maintaining cognitive function and may contribute to AD prevention [23, 27].

This article provides a detailed review of recent research advancements regarding the potential inflammatory link between the skeletal system and AD. It focuses on the shared inflammatory pathways between AD and skeletal health, explores epidemiological connections, and discusses future potential therapeutic approaches.

## 2. Origin of neuroinflammation in AD

Due to the presence of the blood-brain barrier (BBB), the brain has long been considered an immune-privileged organ [28], effectively preventing neuronal peptides from interacting with immune cells and triggering autoimmune responses [29]. However, in many cases, the brain and immune system can communicate bidirectionally [30]. Basic scientists refer to the preclinical phase of AD as the "cellular phase of AD." Changes in neurons, microglial cells, and astrocytes drive the subtle progression of the disease before cognitive impairments are observed [21]. This process begins 20 years before the clinically detectable onset of cognitive impairment and continues until symptoms appear, typically characterized by episodic memory deficits and executive dysfunction [31]. By this point, Aβ deposition has accumulated significantly, nearing its peak. Tau pathology has also begun to accumulate in specific regions of the neocortex a few years before symptoms emerge, and its presence is strongly associated with clinically detectable cognitive impairments and the occurrence and progression of brain atrophy [32].

**Figure 1.**
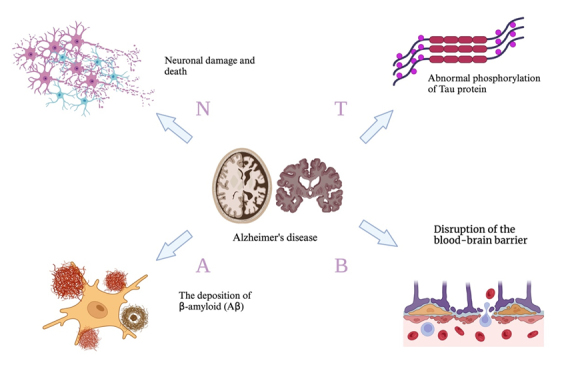
**Major Pathological Features of Alzheimer's Disease and Their Interrelationships This figure illustrates the four core pathological processes underlying AD, including (A) deposition of Aβ, (T) abnormal phosphorylation of Tau protein, (N) neuronal damage and death, and (B) disruption of the blood-brain barrier**. The central structures depict the characteristic brain atrophy and internal anatomical changes observed in AD. These processes collectively interact to drive synaptic dysfunction, network disruption, and the progressive decline in cognitive function. Created by BioRender.

It has been suggested that "X" be incorporated into the A-T-N framework, where "X" represents biomarkers arising from the aforementioned or yet-unrealized pathologies, as well as the dynamic changes occurring as AD progresses. "X" is neither solely upstream nor solely downstream of A/T/N. It encompasses biomarkers that include prominent damage and circulating immune and inflammatory markers [33]. We propose that this "X" corresponds to "B," which refers to the blood-brain barrier, facilitating communication with the peripheral circulatory system and focusing on peripheral inflammatory factors and biomarkers. While Alzheimer's disease is not a normal consequence of aging, age remains its primary risk factor in most cases [34] (as shown in Fig. 1).

Changes in protein aggregation can now be detected in the living human brain using plasma, cerebrospinal fluid (CSF), and neuroimaging biomarkers, which may be valuable for disease staging, predicting outcomes, and assessing the modulation of different treatments [2, 8, 20, 35-39]. Inflammatory-related proteins measured in blood are correlated with overall dementia incidence. Four specific inflammatory proteins have been particularly emphasized for their significant association with the onset of dementia: CX3CL1, EN-RAGE, LAP TGF-β-1, and VEGF-A [20]. In CSF, proteins related to neuro-inflammation, such as NGAL, CXCL-11, sTREM1, and sTREM2, have been shown to correlate with AD pathology [38]. NGAL in CSF may serve as a biomarker for predicting the transition from mild cognitive impairment (MCI) to AD dementia and may reflect synaptic AD pathology [39].

Furthermore, pro-inflammatory proteins like NGAL and CXCL-11 significantly increase concentration during the early stages of the disease and can effectively identify MCI patients. At the same time, the anti-inflammatory molecule sTREM2 reaches its highest concentration in the later stages of the disease, distinguishing AD patients from cognitively healthy controls. More importantly, sTREM2 is significantly associated with the AD group's Tau and phosphorylated form (pTau). These findings suggest that proteins secreted by dysfunctional microglia may have harmful and protective roles in the pathological development of AD [38]. Additionally, the calcium graphite acid (GCA) level in the blood of AD patients is significantly higher than in healthy individuals, which is negatively correlated with cognitive function [40]. Through mass spectrometry analysis, researchers identified 173 protein changes associated with different stages of AD, revealing abnormalities in 17 pathways. By analyzing the 5xFAD mouse model, 15 proteins related to Aβ (such as MDK, NTN1, SMOC1, SLIT2, and HTRA1) were identified, suggesting that this model shares protein characteristics with clinical AD while also showing activation of autophagy and interferon response pathways [36].

In clinical practice, AD is diagnosed using β-amyloid pathology biomarkers, such as low CSF Aβ42 or an increased CSF Aβ40/Aβ42 ratio, and increased tracer retention in amyloid PET scans [37]. Tau pathology biomarkers, such as increased phosphorylated tau in CSF and increased tracer retention in tau PET scans, are also used. Elevated levels of phosphorylated tau in CSF can reflect the early stages of tauopathy. As amyloid deposition begins, soluble monomeric Aβ42 and the Aβ42/40 ratio in CSF and plasma decrease, followed by increases in various forms of p-tau and total tau, which collectively mark the progression of amyloid deposition [2]. As amyloid accumulation becomes widespread, tau pathology begins to spread to the neocortex, and these changes can be detected through amyloid and tau imaging via PET scans [41]. As tau pathology spreads, compounds such as PBR28 can be used in PET scans to detect microglial activation *in vivo* [42].

The main neuropathological features of AD are the accumulation of extracellular Aβ plaques and intracellular neurofibrillary tangles (NFTs) [43]. As the primary component of amyloid plaques, Aβ is generated through sequential proteolytic cleavage of the amyloid precursor protein (APP) by α- or β-secretase (BACE1) and γ-secretase, resulting in either non-amyloidogenic or amyloidogenic processing patterns [44]. After processing in the endosome, Aβ is secreted into the interstitial fluid (ISF) during synaptic activity regulation [45]. The formation of these sediments follows a specific pattern, commencing from the olfactory cortex, subsequently extending to the entorhinal cortex, the CA1 region of the hippocampus, and ultimately reaching the cortical association areas, with particular emphasis on the frontal, parietal, and temporal lobes. Soluble Aβ and insoluble Aβ (e.g., fibrillar aggregates or plaques) differ in triggering neuroinflammation. The former can directly bind to surface receptors on microglial cells, astrocytes, and other glial cells, rapidly activating inflammatory pathways and inducing the production of pro-inflammatory factors and reactive oxygen species, which further increase blood-brain barrier permeability and activate the complement system [5, 46]. The latter, due to its formation of stable deposits in the brain, continuously releases toxic intermediates, providing a sustained stimulus that amplifies the chronic inflammatory response of glial cells and erodes neuronal synapses [47].

Tau pathology, rather than Aβ accumulation, is closely associated with cognitive impairment and regional brain atrophy [54]. Tau is a microtubule-binding protein generally localized to the axons of neurons. In AD, tau becomes hyperphosphorylated and undergoes other post-translational modifications such as acetylation and ubiquitination. These modifications lead to the aggregation of tau into oligomers and paired helical filaments, forming structures observed in the neuronal soma surrounding Aβ plaques (NP tau), nerve cell projections (NT), and neurofibrillary tangles (NFTs) in dystrophic neurites [35].

Tau protein and its complexes with Aβ diffuse through the hippocampal cortex and bind to pattern recognition receptors on microglia and astrocytes [55], triggering inflammatory responses and neurotoxicity [43]. Aβ-induced systemic inflammation, which enters circulation, is one cause of microglial activation. While this activation is destructive, it may also have some reparative effects. Inflammatory regulators, as downstream drivers of Aβ and NFT-induced neurotoxicity, have become important targets for AD treatment [43].

Clinical neuropathological correlation analyses show that tau pathology spreads throughout the AD brain over the years, following a particular distribution pattern within neuroanatomical networks. This pattern forms the basis for Braak staging [56]. Although several patterns can be observed, the most common involves tau pathology first appearing in the entorhinal cortex (Braak I-II), which is seen in most individuals over 60, both with and without AD. Tau pathology then spreads to the lower temporal neocortex and limbic structures (Braak III) a few years before the onset of cognitive decline. These regions are involved in memory formation and visual object recognition functions. Subsequently, tau pathology appears in the amygdala and thalamic areas, which are involved in emotional processing and memory (Braak IV). Finally, as cognitive decline progresses, tau pathology emerges in other areas of the neocortex (Braak V-VI) [57].

### 2.1 Transitional activation of microglia and astrocytes

Neuroinflammation is a complex and multifaceted response of the central nervous system (CNS) to injury, infection, or disease, playing a key role in the pathogenesis of AD [58, 59]. The emerging roles of "metabolic inflammation" and stress in AD pathogenesis have been increasingly recognized [60]. This process is characterized by activating resident immune cells, primarily microglia and astrocytes, which produce inflammatory mediators [61]. These mediators can lead to neuronal damage and loss [62].

### 2.1.1 Microglia

The microglial dysregulation hypothesis suggests that the root cause of AD may lie in the abnormal activation of microglia, leading to increased cytotoxicity, which in turn triggers neuroinflammation and functional impairments [63]. This activation dysfunction could be induced by environmental factors and aging, further exacerbating the generation and accumulation of Aβ [64]. Chronic neuroinflammation is considered one of the significant pathophysiological features of AD. Microglial proliferation, differentiation, and survival depend highly on the CSF-1 signaling pathway. The CSF1 receptor (CSF1R), or the M-CSF receptor, is essential for microglia's growth, differentiation, and survival in the central CNS and monocyte/macrophage lineages [65]. This pathway can be modulated by inhibiting the CSF-1R receptor. Brief depletion of microglia reduces neuroinflammation and improves behavioral test outcomes [66]. Aβ interacts with microglia through TLR3 [67], TLR4, and the NLRP3 inflammasome [68], triggering the release of TIRAP/MyD88, activating the TRAF6 pathway, and leading to the activation of the transcription factors NF-κB and AP-1. This activation results in microglial cell activation, which releases pro-inflammatory factors such as IL-1β and TNF-α [69].

Aβ can inhibit the nuclear translocation of the transcription factor EB (TFEB) in microglia, impairing lysosomal acidification and affecting Aβ degradation [70, 71]. Aβ induces inflammatory processes due to its accumulation in the brain and ability to bind to the complement system's C1 component. The complement cascade is triggered by the C1q complex, which is strongly expressed by microglia and enriched in the plaque cell microenvironment, thus inducing neuroinflammation [72]. In AD patients, several complement components (C1QA, C1QB, C1QC, and CLU) and the TREM2 adapter protein TYROBP are enriched around amyloid plaques [72, 73]. Lack of C1q, C3, or CR3 reduces synaptic loss and improves cognitive function in AD mouse models [74, 75]. Along this pathway, molecular changes before neurodegeneration are induced by tau accumulation in hippocampal synapses can be rescued by blocking C1q. Additionally, in tau pathology, abnormal microglial phagocytosis is triggered by synapses marked by C1q [76].

### 2.1.2 Astrocytes

One of the pathological features of AD is the proliferation of reactive astrocytes, which is closely associated with neuronal loss and cognitive impairments [77]. In a triple-transgenic AD mouse model harboring APPswe, presenilin 1, and tauP301L mutations, comprehensive atrophy of hippocampal astrocytes precedes the proliferation of astrocytes associated with Aβ plaques [62]. This suggests that neuroinflammation is not only a result of AD but may also actively promote its development and progression. Furthermore, chronic neuroinflammation, characterized by sustained microglial activation and continuous exposure to pro-inflammatory cytokines, can lead to functional and structural changes in neurons, ultimately resulting in neuronal degeneration [59].

Alterations in cholesterol metabolism within the brain also play a significant role in AD, with changes in cholesterol oxidant levels closely related to disease progression. Astrocyte-conditioned medium analysis reveals that cholesterol oxidants significantly increase the release of lipocalin-2 (LCN2), cytokines, and chemokines. Neurons co-cultured with astrocytes treated with cholesterol oxidants significantly reduce PSD95 protein levels, while cleaved caspase-3 levels significantly increase [77]. Astrocytes in the AD brain significantly increase in α2-Na+/K+ ATPase (α2-NKA) activity. Inhibition of this enzyme's activity significantly reduces neuroinflammation and brain atrophy.Additionally, reducing the expression of α2-NKA prevents the accumulation of tau protein in AD model mice, further emphasizing the role of this enzyme in AD. α2-NKA exacerbates neuroinflammation in AD by modulating the pro-inflammatory protein Lcn2. These studies highlight the importance of reactive astrocytes in the pathological mechanisms of tau-related diseases in mice and identify α2-NKA as a key factor in regulating neuroinflammation [78]. Aβ and ApoE are upstream of neuroinflammation induced by sulfatase deficiency, suggesting a positive feedback loop where increased ApoE expression may amplify sulfatase loss. The proliferation of astrocytes and upregulation of ApoE triggered by CNS sulfatase deficiency is not a secondary response to microglial proliferation. Still, it is driven by activating the STAT3 and PU.1/Spi1 transcription factors [79]. Astrocytes and microglia communicate via secreted proteins (such as C1Q and APOE) and receptors (such as TREM2 and the associated TYROBP), which overall lead to astrocyte activation (GFAP, C4, Clu, Prdx6, Cst3, and Serpina3) near amyloid plaques. This response results in inappropriate regulation of the classical complement cascade [21, 72].

TNF-α and IL-6 have been found to increase the activity of BACE1 and the expression of NFκB, leading to Aβ production in the AD brain. After interacting with microglia and astrocytes, Aβ triggers the migration of other inflammatory molecules to the site of inflammation through chemotaxis, further exacerbating neuro-inflammation [80].

## 3. Bone health and inflammation

### 3.1 Inflammatory senescence leads to alterations in the bone marrow microenvironment

As individuals age, significant changes occur within the skeletal microenvironment that can substantially affect bone health [81]. Notably, bone marrow mesenchymal stem cells (BMSCs) exhibit a growing inclination toward adipocyte differentiation rather than osteogenic activity, impacting both their quantity and functionality. Concurrently, there is an increased rate of apoptosis among osteoblasts and osteocytes, which results in a diminished population of bone-forming cells. Moreover, osteoclast activity has been observed to heighten, enhancing bone resorption. The structural integrity of osteocyte components may also decline with age. At the same time, reductions in H-type blood vessels, alongside an accumulation of adipose tissue within the bone, may further compromise structural support. Despite these challenges, a comprehensive understanding of these changes allows us to develop targeted strategies to promote healthier bone outcomes as we age. By emphasizing bone health, we can enhance overall well-being and vitality, improving quality of life.

As age increases, the risk of DNA damage escalates, with telomere shortening being one contributing factor [82]. Additionally, mitochondrial oxidative stress produces reactive oxygen species (ROS), and chemical damage is also a contributing factor [83, 84]. Upon activation of sensor proteins (ATM, ATR) [85], “cell cycle brake” genes (p53, p21, p16, Rb) play a critical role in determining whether a cell enters senescence arrest [86]. Cellular senescence (CS) is a form of cell cycle arrest that occurs during normal aging to prevent the proliferation of damaged cells [87]. This process involves a series of phosphorylations that transmit signals to the transcription factor NF-κB, triggering and maintaining the secretion of a wide range of pro-inflammatory factors (IL-1, IL-6, TNF-α, TNF-β, PGF2α), proteases, and chemokines. These are referred to as the "senescence-associated secretory phenotype" (SASP), which disrupts the bone microenvironment [88, 89]. The release of inflammatory factors in the bone leads to the polarization and infiltration of macrophages, which further release related inflammatory factors to amplify this effect, playing a role in immune regulation [90]. This forms an amplified feedback loop of "immune-bone" interactions [91]. Endothelial cells are also activated, stimulating the local vascular endothelial cells to express more adhesion molecules (ICAM-1, VCAM-1), accelerating the migration of peripheral immune cells from the blood to the bone marrow and bone tissue [92]. This alteration in the microenvironment disrupts bone metabolic homeostasis, leading to osteoporosis [27].

### 3.2 The effect of inflammation on bone metabolism

The RANKL/RANK/OPG axis plays a key role in osteoporosis [93]. RANK ligand (RANKL) is an essential mediator of osteoclast formation, function, and survival, produced not only by bone marrow stromal cells and osteoblasts but also by T cells. RANKL binds to the RANK receptor, which is expressed by monocytes/macrophages (Mo/Mø), osteoclast precursors, dendritic cells (DCs)/antigen-presenting cells (APCs), and T cells. Mature osteoblasts and B cells express the RANKL antagonist and TRAIL-binding factor OPG and inhibit RANKL-induced osteoclastogenesis [94]. In the inflammatory infiltrated bone microenvironment, some macrophages with a pro-inflammatory phenotype not only enhance RANKL expression and inhibit OPG production, thereby reducing the number of RANK receptors bound by OPG but also induce osteoblast apoptosis or functional decline. As a result, the bone resorption pathway is persistently activated, while the bone formation pathway is significantly weakened [95]. Moreover, in the presence of continuous aging and inflammation, the number or function of M2 macrophages is relatively insufficient, making it difficult to counteract the destructive effects of M1 macrophages [96].

Energy depletion-induced AKT inhibition triggers an inflammatory signaling cascade, leading to glucose deprivation, endoplasmic reticulum stress, and the production of ROS. If HIF-1α and AMPK cannot restore AKT and subsequent glucose uptake, inflammasome formation and IL-1β synthesis attempt to recover this uptake [97]. Endothelial cell disruption, on one hand, leads to the systemic spread of inflammatory factors from the local microenvironment, causing systemic inflammation, and on the other hand, amplifies the synergistic effects of immune cells in the vasculature. Th17 cells secrete IL-17 and are highly associated with bone destruction [98, 99]. IL-17 cooperates with RANKL signaling to further promote osteoclast formation and activity. Some B cells may directly differentiate into osteoclast precursors, further enhancing bone resorption potential. Osteoblast function is significantly impaired in a high-inflammatory environment, with reduced expression of osteocalcin and type I collagen [100]. Genetic factors associated with osteoporosis susceptibility in the Chinese Han population have been identified, including rs10490571, rs956730, and rs3917225 in IL-1R1 and rs17042888 in IL-1RN. These findings deepen the understanding of the role of IL-1R1 and IL-1RN in the genetic etiology of osteoporosis, though they need to be validated in larger cohorts [101]. Osteoprogenitor cells (bone marrow stromal stem cells) tend to differentiate into adipocytes rather than osteoblasts. Osteocytes' network structure is also damaged, weakening their ability to sense mechanical forces and regulate bone remodeling. The lack of mechanical stimulation leads to reduced osteoblast activity, decreased bone formation, and reduced bone density [102].

Macrophages regulate osteocytes, which secrete inflammatory mediators to control the coupling of bone resorption and formation [103]. The intensification of bone resorption, impaired bone formation, and continuous decrease in bone density eventually trigger or accelerate the risk of osteoporosis and fractures. This creates a vicious cycle of "inflammatory amplification" and "aging exacerbation."

### 3.3 Inflammatory response after fracture

Inflammatory cytokines, autoantibodies, and multiple signaling pathways (such as RANKL, Wnt signaling, and the IL-17/23 axis) play crucial roles in bone injury [104]. During primary bone development and fracture healing in adults, the formation of new blood vessels is essential [105, 106]. Reduced or insufficient blood flow is associated with impaired fracture healing and conditions such as osteoporosis and other low bone mass diseases. Angiogenesis precedes osteogenesis, providing essential nutrients, growth factors, and oxygen necessary for the formation and differentiation of osteoblasts and osteoclasts [107]. Furthermore, interactions between bone marrow endothelial cells and hematopoietic progenitor cells, mediated by molecules such as VCAM-1, VLA-4, and the chemokine SDF-1, are critical in angiogenesis [108]. These interactions are particularly relevant in fracture healing, where angiogenesis is a key component of the repair process [105, 106].

## 4. Studies on the correlation between AD and bone health problems

### 4.1 Bone density and AD

A significant association exists between BMD and AD [109-112]. This association is apparent in the late stages of the disease and its early phases. In the subclinical stage, comorbidity between AD and osteoporosis is already evident, manifested by increased bone resorption, enhanced bone remodeling, and osteophagy activity. However, it is not related to vitamin D status [113].

The aberrant immune mechanisms in OP involve pro-inflammatory cytokines and mediators at a chronic pathological level that promote bone resorption through osteoclast differentiation and activation, enhanced RANKL expression, and osteoblast survival inhibition. The inflammatory milieu generated by macrophages, dendritic cells, and local fibroblasts stimulates the proliferation of Th17 cells, which subsequently express RANKL, thereby promoting the upregulation of RANKL expression in osteoblasts and fibroblasts and exacerbating the M1 polarization of macrophages [114].

Research has shown that AD patients have twice the probability of developing osteoporosis and fractures compared to non-AD patients [115]. Between 2002 and 2005, a study of over 3,600 participants found that individuals with lower BMD, particularly in the femoral neck and throughout the body, were more likely to develop dementia. This suggests that BMD could potentially serve as an early predictor of dementia, though further validation is needed [110]. Reduced BMD is associated with decreased volume of specific brain regions, especially those typically affected by AD. This association remains significant even after controlling age, gender, and cognitive function scores [116]. Furthermore, a systematic review comparing BMD in elderly AD patients and those without dementia found that AD patients exhibited lower BMD in the femoral neck and other sites, including the body, trunk, femur, and lumbar spine [111].

In a study of 78,994 osteoporosis patients and an equal number of controls from Korea, the incidence of AD and Parkinson's disease (PD) was significantly higher in the osteoporosis group compared to the control group. After adjusting for various factors, the incidence rates of AD and PD were 1.27 and 1.49 times higher, respectively [117]. Hip BMD was generally lower in dementia patients [112, 118]. In early AD patients, bone metabolism biomarkers and BMD were significantly lower than in healthy controls, suggesting that early screening for AD could be facilitated by monitoring BMD [119]. Further, calcaneal BMD was found to have a statistically significant causal effect on AD risk, supporting its role as a risk factor for AD, and highlighting the need for more in-depth investigation into the potential mechanisms linking the two [120].

A total of 42 male patients with early AD and 40 age-matched healthy elderly volunteers were included in the study. The association between early AD and bone density was assessed, revealing that patients with early AD exhibited significantly higher levels of urinary DPD/Cr, urinary Ca/Cr, and serum osteocalcin compared to the healthy control group. BMD data indicated that the cortical and total BMD at 38% of tibial length in early AD patients was lower than that of the healthy control group. Furthermore, the Montreal Cognitive Assessment scores negatively correlated with serum osteocalcin and urinary DPD/Cr levels. Abnormal levels of urinary DPD/Cr, urinary Ca/Cr, and cortical BMD were identified as independent risk factors for male patients with early AD [119].

Recent meta-analyses and systematic reviews on the relationship between BMD and AD have provided essential insights [121, 122]. A recent study analyzing multiple databases, including 136,222 participants, revealed that the risk of cognitive impairment was significantly higher in osteoporosis patients. Despite significant heterogeneity in the meta-analysis, subgroup analyses identified potential sources of variability across different study types, age groups, and outcome measures. The baseline BMD value was significantly associated with the incidence of dementia, with higher BMD being associated with a reduced risk of dementia. These findings underscore the importance of BMD in the risk assessment of dementia, particularly AD [122]. It has been hypothesized that bone loss may represent an early precursor of AD, mediated through dysfunction in central serotonergic pathways [123]. In a mouse model expressing human tau protein, pathological tau phosphorylation was observed in serotonin-producing neurons in the brainstem, and these mice exhibited a higher frequency of bone loss even before significant damage occurred. In AD, hypothalamic atrophy has been associated with osteoporosis, concluding that osteoporosis may be related to neurodegenerative changes in the hypothalamus. Specifically, AD patients experience an increase in leptin levels within two years, and this increase in leptin is associated with hypothalamic atrophy. However, no significant associations were found between changes in leptin or insulin-like growth factor-1 levels and hypothalamic atrophy or osteoporosis [124].

Contradictorily, a two-sample Mendelian randomization (MR) study exploring the relationship between osteoporosis and AD found no significant causal link. Sensitivity analyses also supported this conclusion [125]. Additionally, another study examining the relationship between schizophrenia, bipolar disorder (BD), and AD with BMD found no significant causal relationship between the risk of these mental disorders and changes in BMD. Finally, a study investigating the relationship between five psychiatric disorders (including schizophrenia, depression, AD, Parkinson's disease, and epilepsy) and the risk of osteoporosis did not find a significant causal relationship, despite correlations observed in observational studies, suggesting the need for further research to elucidate the potential mechanisms [126].

### 4.2 Fractures and AD

Osteoporosis is a well-known risk factor for fractures [127], and bone loss is linked to subsequent cognitive decline, further emphasizing the interconnection between these diseases [40]. In AD patients, lower bone density is associated with an increased fracture risk, which may be related to dysregulation of sympathetic neurons [128]. AD patients are more than twice as likely to experience fractures compared to non-AD patients, with this increased risk being observed within the first year after an AD diagnosis [129, 130]. A study conducted in the UK extended these findings, reporting that AD patients had a threefold higher incidence of hip fractures at any given time point compared to those without AD [131]. This increased risk is not limited to the period immediately following AD diagnosis but persists throughout the course of the disease. Moreover, a higher post-fracture mortality rate is observed in AD patients, with 27.2% of AD patients and 13.6% of non-AD patients failing to survive beyond six months after a fracture [131].

An observational study involving 2,041 women assessed for peripheral fragility fractures also examined the prevalence of Alzheimer's disease and other dementias (ADD), comparing it with the French population. The results showed that among the respondents who had an average age of 85 years, the prevalence of ADD was 13.5%. As ADD patients tend to be older, the prevalence significantly increased with age: 0% in those under 55, 1.8% in those aged 55-74, 13% in those aged 75-79, and 29.7% in those aged 85-89. Proximal femoral fractures were the most common (77%), followed by wrist fractures (13%) and proximal humeral fractures (10%) [132]. The study concluded that the prevalence of ADD in this group was 3-4 times higher than in the general French population, with a significant increase with age. The conclusion highlighted those postmenopausal women with severe osteoporosis, especially those with hip fractures, have a higher prevalence of ADD and thus require effective care for this high-risk group [132]. In AD patients, in addition to traditional cognitive impairment, atypical clinical features often affect quality of life [1]. Damage to visual-spatial abilities, executive function, and motor control usually leads to falls and gait instability, increasing the risk of fractures [129]. MRI studies have shown that generalized brain atrophy and white matter hyperintensities are associated with a decline in gait scores, which include various parameters such as rhythm, speed, and variability. This suggests that cognition is non-specific in gait function [133]. The basal nucleus of Meynert, a major provider of cholinergic signals to the cortex, is believed to be crucial in revealing the relationship between AD and gait dysfunction. Rehabilitation therapy has been shown to affect cognitive function and brain activity in elderly patients hospitalized for fractures caused by falls. Researchers used EEG and dementia rating scales to assess patients' cognitive function and brain activity before and after rehabilitation therapy. The results indicated that while there were no significant differences in overall cognitive function before and after rehabilitation, memory function did improve. Furthermore, after rehabilitation, brain activity patterns became more normalized [134].

**Figure 2.**
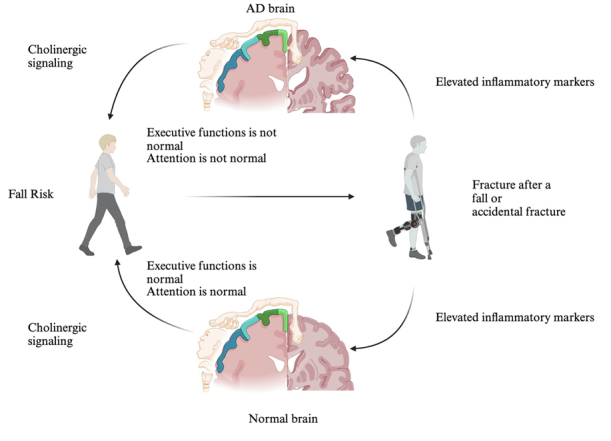
**Cholinergic Dysfunction, Inflammation, and Cognitive Impairment Contributing to Fall Risk and Fracture in Alzheimer’s Disease A schematic comparing normal brain function to AD pathology, illustrating how deficits in cholinergic signaling, impaired executive functions, and reduced attentional capacity can collectively increase fall risk**. Elevated inflammatory states in AD further exacerbate neuromuscular and cognitive challenges, rendering individuals more susceptible to fractures after fall or accidental injuries. Conversely, a normally functioning brain with intact cholinergic signaling, executive function, and attention demonstrates a lower overall risk of falls and associated complications. Created by BioRender.

Post-operative delirium (POD) after skeletal surgery has been associated with an increased risk of developing dementia within three years following surgery [135]. Inflammatory markers such as C-reactive protein (CRP) and IL-6 are commonly elevated after fractures, and these increases are associated with an increased risk of all-cause dementia [136]. While these markers are not specific to AD, peripheral inflammation has also been linked to AD [137] as shown in Figure 2.

### 4.3 Osteoarthritis and AD

Chronic pain caused by osteoarthritis (OA) is associated with impairments in multiple cognitive domains, including memory, attention, processing speed, and executive function, suggesting that chronic pain plays a critical role in the impact of OA on clinical symptoms of AD [138]. New evidence suggests that OA and its associated symptom burden may increase the risk of Alzheimer's disease and related dementias (ADRD) [138]. The association between OA and increased ADRD risk is particularly pronounced in individuals with both OA and pain, with mood disorders potentially mediating this relationship [138]. Using cross-sectional data representative of U.S. adults aged 65 and older, the relationship between OA, pain intervention, and ADRD was assessed. Regardless of OA status, adults with PI had a significantly higher likelihood of ADRD compared to those without OA or PI. Even after adjusting for various confounding factors, the relationship between PI and ADRD remained significant and positive in individuals with and without OA [139]. In an AD mouse model, through the analysis of brain tissue and neuroinflammation, knee OA accelerated amyloid plaque deposition and neurodegeneration in AD-OA mice, indicating that OA is a risk factor for AD [140]. A recent study explored whether OA affects hippocampal volume (HpVR) change over time in cognitively normal older adults. The researchers used data from the Alzheimer’s Disease Neuroimaging Initiative (ADNI) on cognitively normal (NC) individuals to examine the cross-sectional and longitudinal relationships between OA and HpVR [141]. Compared to individuals without OA, those with OA showed a faster rate of hippocampal volume reduction after controlling for other potential confounding factors, including age, education, sex, and APOE4 genotype. In summary, OA status was significantly associated with the change in HpVR over time in cognitively normal individuals [141].

Clinical trials in OA patients using nerve growth factor (NGF) inhibitors have shown that NGF inhibition may adversely affect joint structure. It has been reported that during OA, the nerve growth factor receptor (NGFR) is upregulated in bone cells and plays a critical role in the remodeling and repairing OA joints. Specifically, NGFR is expressed in chondrocytes but not in osteoprogenitor cells, and it is induced by TNFα to mitigate NF-κB activation while maintaining proper BMP-SMAD1 signaling and inhibiting RANKL expression in mice. NGFR deficiency results in the hyperactivation of NF-κB in mouse OA joints, impairing bone formation and enhancing bone resorption, reducing subchondral bone and osteophyte formation. In human OA cartilage, NGFR expression negatively correlates with NF-κB activation [142].

### 4.4 Rheumatoid arthritis and AD

Under inflammatory conditions in rheumatoid arthritis (RA), several molecules such as IL-1β, IL-6, TNF-α, IL-17, and hypoxia-inducible factor-1α (HIF-1α) are produced, which can mediate bone loss [143, 144]. Independent predictors of BMD in RA include sclerostin (SOST) and C-terminal telopeptide (βCTX), while in AS, CRP serves as an independent predictor [145]. RA, psoriasis, ankylosing spondylitis, inflammatory bowel disease, ulcerative colitis, and Crohn's disease are all associated with an increased risk of AD [146]. RA is an independent risk factor for osteoporosis [147]. In arthritis, systemic inflammation and disease activity may drive the onset of vascular and bone diseases [148]. Decreased BMD and tendon abnormalities (such as tendosynovitis and enthesitis) are common comorbidities in RA patients [149]. In summary, compared with healthy controls, RA patients show significant cognitive decline, and the incidence of dementia due to AD increases [150].

In RA, amyloid substances triggered by immune system activity impact the degradation of bone joint structures, with the blood-brain barrier mediating this relationship between the two [151]. Some genes associated with increased inflammation risk have also been found to be linked with an increased risk of Alzheimer's disease [152]. Notably, the transcriptional expression of HLA-DRA and IPMK in brain tissues of AD patients differ significantly from that in healthy individuals. The incidence of AD in RA patients is substantially higher than in those without RA, but RA patients receiving anti-TNF-α treatment show a lower risk of AD. This finding suggests that anti-TNF-α therapies may serve as a potential treatment strategy for Alzheimer's disease [153]. RA, as a chronic autoimmune disease, is treated with medications that also show protective effects for dementia patients. However, observational studies exploring the relationship between these two diseases have yielded conflicting results, with no significant association identified [154]. Possible reasons for these inconsistencies include the use of RA medications, sample selection biases, and differences in RA diagnostic criteria [155]. Although TNF inhibitors may be associated with a lower ADRD risk in patients with cardiovascular disease, no apparent risk-reducing effect has been observed overall. Statistical analysis reveals no significant association between ADRD risk and the use of the medications above [156].

Despite early research on the potential link between these two diseases, related findings remain controversial [157]. Using big data analysis from Taiwan's 2005 Long-term Health Insurance Database, case-control studies have investigated the relationship between early RA and AD. These results show a significant inverse association between early RA and AD, even after adjusting for potential confounding factors [157]. Some controversial studies suggest a reverse relationship between AD and RA, indicating that individuals with RA might have a reduced risk of developing AD, and vice versa [158]. A population-based retrospective cohort study conducted from 1980 to 2009 in Olmsted County, Minnesota, using the ACR 1987 criteria to diagnose newly diagnosed RA patients, found that the risk of dementia in RA patients appears to decrease over time, especially compared to the general population [159].

### 4.5 Periodontitis and AD

Dysregulation of immune system balance is one of the shared mechanisms between periodontitis and AD. Upon immune system activation, CD4^+^ T cells can differentiate into subtypes, including Th1, Th2, Th17, and regulatory T cells (Tregs). These subtypes play distinct roles in the immune response, with Th1 and Th17 cells primarily mediating inflammatory responses, while Th2 and Tregs exert anti-inflammatory effects [160]. In AD model mice (APP/PS1), the proportions of Th1 and Th17 cells increase while the proportion of Th2 cells decreases. This immune imbalance may exacerbate the pathological progression of AD [160]. Periodontitis may lead to an increase in Aβ-reactive Th1 and Th17 effector T cells (Teffs), and the migration of these cells accelerates behavioral and pathological changes in AD mice. Therefore, systemic inflammation caused by periodontitis may influence the progression of AD by promoting Aβ deposition and neuroinflammation [160].

Chronic inflammatory diseases, such as periodontitis, have a pathological link with AD. IL-1β and TNF-α are the most important and earliest cytokines upregulated following periodontal infection. Patients with chronic periodontitis also have a higher risk of developing AD [161]. The systemic upregulation of these two cytokines promotes a pro-inflammatory environment in the brain. Systemic inflammation caused by periodontitis also enhances brain inflammatory responses, subsequently exacerbating the pathology and cognitive impairment in 3×TG-AD mice [162]. Periodontitis may promote the onset and progression of cognitive impairment by modulating the balance of Th17/Treg both within and outside the brain [163].

The sustained release of these pro-inflammatory cytokines may impact the brain like systemic inflammation [164]. This may be due to the infiltration of inflammatory factors disrupting the blood-brain barrier. *Porphyromonas gingivalis* secretes Porphyromonas gingivalis-derived peptidylarginine deiminase (PPAD), which can convert arginine to citrulline, potentially triggering a systemic inflammatory response and is associated with the pathogenesis of AD [161]. Additionally, the excitotoxic toxin quinolinic acid (QUIN) is produced by activated monocytes and can induce the expression of IL-1β in astrocytes, thereby promoting the development of neuroinflammation [165].

## 5. Risk factors common to both scenarios

### 5.1 lifestyle

The study assessed the association between 25-hydroxyvitamin D (25(OH)D) blood levels and brain structural changes. The results showed that low levels of 25(OH)D were associated with reduced brain volume in the overall study population's right olfactory and straight muscle gray matter regions. In AD participants, positive correlations were found between 25(OH)D levels and the left hippocampal gyrus, amygdala, and hippocampal regions. Low blood 25(OH)D levels were associated with reduced volumes of the olfactory and hippocampal areas of elderly individuals with cognitive decline [150].

At the animal level, a vitamin D-deficient diet was provided to induce vitamin D deficiency in 5xFAD mice, and changes in the mRNA levels of genes related to Aβ processing were observed. The results showed an increased Aβ load in the brain [166]. 23R,25-dihydroxyvitamin D3 and 3-epi-25-hydroxyvitamin D3 metabolites were significantly elevated in the plasma of AD patients [167]. The active form (1α,25-dihydroxyvitamin D3) showed significant differences between health and disease states. However, no significant correlation was found between total circulating forms and total active forms in either the disease or healthy groups [167]. A prospective cohort study explored the long-term relationship between vitamin D status and dementia risk. An 18-year follow-up found no significant association between plasma 25(OH)D concentrations, dietary vitamin D intake, or vitamin D synthesis genetic risk score (GRS) and the risk of all-cause dementia, Alzheimer's disease, vascular dementia, or cognitive decline [168].

Toxic metals, including cadmium, lead, aluminum, and arsenic, can accumulate in the bones through external intake and potentially replace calcium in hydroxyapatite, the primary mineral component of bones. These metals, in particular, hurt bone mineral density [22].

### 5.2 Genetic predisposing factors

The APOE-ε4 gene variant is closely associated with the risk of AD, but it is not the only pathogenic factor [16]. Macrophages derived from the bone marrow can effectively clear β-amyloid plaques in the brain, and this mechanism is closely linked to the type of apolipoprotein E (APOE) expressed [17]. Studies on the APOE4 allele show it hurts microglial cells' AD pathology [169, 170]. By removing APOE4 from microglial cells, researchers have found that neuroprotective responses improve, and pathological changes in AD mice are reduced [170]. Macrophages expressing ApoE2 demonstrate greater efficiency in degrading β-amyloid plaques [17].

A study recruiting 120 participants found that a higher ratio of visceral adipose tissue (VAT) to subcutaneous adipose tissue (SAT) (VAT/SAT) was significantly associated with increased amyloid deposition in the right precuneus cortex, with this association being limited to males. Further analysis revealed that a higher VAT/SAT ratio, body mass index (BMI), and insulin resistance were closely related to reduced cortical thickness in Alzheimer’s disease (AD)-related cortical regions, particularly in the temporal lobe. Additionally, the level of amyloid deposition in the right precuneus cortex was also significantly correlated with the decrease in cortical thickness in the AD-related cortical regions. Future larger-scale longitudinal studies are needed to validate these findings and establish causal relationships [171].

TREM2 is primarily expressed in microglial cells of the central nervous system, osteoclasts in bone tissue, and macrophages and other myeloid cells throughout the body [172, 173]. TREM2 is a 230-amino acid glycoprotein that belongs to the immunoglobulin (Ig) superfamily. Functionally, TREM2 inhibits the expression of cytokines and chemokines as a negative regulator of Toll-like receptor (TLR)-mediated responses and promotes the phagocytic activity of microglial cells [174]. The TREM2-DAP12 signaling pathway plays a vital role in regulating the inflammatory response of microglial cells. Its activation can suppress excessive inflammation, prevent neuronal damage, and promote the production of therapeutic cytokines. In diseases like Alzheimer's disease, the lack of DAP12 and dysfunction of TREM2 lead to a reduced ability of microglial cells to clear β-amyloid plaques, accelerating neurodegeneration and worsening the disease's pathological progression [175]. Microglial cells with TREM2 gene knockout show decreased internalization of LDL and CLU [176]. β-amyloid plaques bind to lipoproteins, and this complex can be efficiently engulfed by microglial cells in a TREM2-dependent manner [175]. Heterozygous mutations in the TREM2 gene impair microglial function and accelerate the onset of late-onset AD, affecting the wound-healing markers in astrocytes and the myelination in oligodendrocytes, with vascular abnormalities seen in pericytes. Macrophages in humans carrying the TREM2 AD variant have a reduced ability to take up the Aβ-lipoprotein complex. The TREM2 deficiency is linked to both AD risk and bone resorption. This signaling pathway is crucial in microglial cells and osteoclasts, connecting neuroinflammation with bone metabolism [177]. Genetic variants of TREM2, such as TREM2 R47H, have been confirmed to increase the risk of developing AD, potentially promoting its progression through enhanced oxidative stress and inflammation [178].

Nasu-Hakola disease (NHD), also known as polycystic lipomembranous osteodysplasia with sclerosing leukoencephalopathy, is a rare autosomal recessive disease characterized by progressive early-onset dementia and the formation of multiple bone cysts. It is primarily caused by loss-of-function mutations in DAP12 or TREM2 [179].

Higher APOE-ε4 allele carriage rates have been observed in patients with behavioral-type Alzheimer's disease compared to those with executive-function-type AD. Among behavioral-type AD patients, 59.5% carry at least one APOE-ε4 allele, while the carriage rate in executive-function-type AD patients is 40%. More than half of both behavioral and executive-function-type AD patients have a family history of dementia or psychiatric disorders, suggesting a potential genetic link to these subtypes of Alzheimer's [180].

In studies on posterior cortical atrophy (PCA), multiple genetic loci are related to PCA. Among them, the APOE gene's proxy SNP rs2075650 on chromosome 19 has been confirmed as an essential risk factor for PCA [181]. Other related loci include rs3818361 on chromosome 1, associated with the CR1 gene, rs3764650 on chromosome 19, related to ABCA7, and rs744373 on chromosome 2, linked to the BIN1 gene. Additionally, three new potential risk loci have been discovered: rs2525776 on chromosome 7, associated with SEMA3C, rs76854344 on chromosome 2, linked to CNTNAP5, and rs72907046 on chromosome 6, related to FAM46A. These findings provide important clues for understanding the genetic basis of PCA and its relationship with AD [182].

Variants of the PS1 (presenilin 1) gene lead to improper folding and aggregation, triggering Alzheimer's disease. Specific mutations, such as P264 and P267 in PS1, have been associated with AD and can offer opportunities for early screening in at-risk populations. These mutations could serve as biomarkers for identifying potential AD patients, enabling early intervention [414].

The relationship between computer-based EEG activity and the PICALM and CLU alleles, which are thought to have risk or protective effects on Alzheimer's disease (AD) patients and health controls, was investigated in a study involving 18 AD patients and 12 controls carrying the PICALM and CLU risk alleles, as well as 35 AD patients and 12 controls with protective alleles. The study calculated the relative power (RP) of conventional EEG frequency bands (delta, theta, alpha, beta, gamma) to quantify brain activity at the source level, as well as the spatial entropy (SE) to describe the regional distribution of RP values across the entire brain. Based on genotype, significant differences in the global RP and SE in the beta frequency band were observed in the AD group. Furthermore, in the 68 cortical regions analyzed in AD, 58 areas showed statistically significant differences in RP. No such significant differences were found in any frequency bands in the control group. These results suggest that the PICALM and CLU genotypes, which are associated with AD, are linked to disruptions in beta dynamics, possibly related to changes in β-amyloid plaques and neurotransmitter metabolism [183].

Cyclophilin B, a chaperone protein residing in the endoplasmic reticulum (ER), plays a crucial role in the correct folding of PS1. This process could represent a novel target for AD treatment. Enhancing or restoring the function of such chaperone proteins may prevent or slow the onset and progression of AD.

Single nucleotide polymorphism (SNP) rs11136000 in the CLU gene has been associated with AD risk in individuals of European ancestry, with the C allele of rs11136000 linked to increased susceptibility to AD [184]. Interactions between the CLU and MS4A4E genes have been observed, demonstrating significant effects, especially in individuals without the APOE ε4, APP, or TREM2 risk variants. If the CLU-MS4A4E risk alleles are absent, the incidence of Alzheimer's may decrease by 8% [185]. In Asian populations, the rs2279590 polymorphism has shown a significant association with AD, with no significant heterogeneity between Asian and European populations [186].

Researchers have studied the three gene polymorphisms of TGF-beta1 in 678 AD patients and 667 controls to explore their potential impact on disease onset. The study also examined Aβ deposition in the brains of 81 AD patients [187]. However, the results did not show any significant changes in genotype and haplotype distribution between AD patients and controls, nor did they find any notable effect on Aβ accumulation. These results do not support the idea that genetic variation at the TGF-beta1 locus influences Alzheimer's disease risk. However, selective induction of TGF-beta2 might occur in NDAD and FAD-14 cases [188].

### 5.3 Hormonal predisposing factors

Osteoporosis has diverse etiologies, primarily related to age, gender, hormone levels, nutritional status, lifestyle, and genetic factors. Specifically, estrogen deficiency is considered one of the leading causes of postmenopausal osteoporosis in women, often referred to as post-menopausal osteoporosis (PMOP).

Both AD and osteoporosis disproportionately affect women, particularly postmenopausal women. Two-thirds of AD patients are women, and studies have shown that the incidence of AD is higher in women compared to men of the same age group, with the most significant differences observed in individuals over 90 years old [189, 190]. Similarly, osteoporosis is a significant health issue for postmenopausal women, as bone loss occurs at a rate of 3-5% within 5-10 years after menopause [191]. The incidence of osteoporosis in women roughly doubles every five years, reaching 50.3% in women aged 85 and older. A common link between these two conditions is the role of estrogen, a group of hormones that decline after menopause, with estradiol being the most potent form. These hormones affect cognitive function and bone health [192, 193]. Estrogen deficiency also increases pro-inflammatory molecules, such as IL-1, IL-6, and TNF-α. These molecules are known to promote the activation of T cells, which in turn induce osteoclast formation, leading to bone loss in osteoporosis [194]. Elevated peripheral concentrations of these cytokines have also been observed in AD patients, linking the two diseases through a shared inflammatory response [14].

Estrogen receptors are highly expressed in the brain. In animal models, estrogen has beneficial effects on brain tissue by promoting the growth of cholinergic neurons and the metabolism of APP [189]. Studies have shown that higher circulating estrogen concentrations, particularly estradiol, are associated with a lower risk of cognitive decline in postmenopausal women [195, 196]. This relationship is modulated by the concentration of sex hormone-binding globulin (SHBG). SHBG binds strongly and specifically to estradiol, reducing its ability to bind to receptors and initiate responses. Research indicates that AD patients have significantly higher SHBG levels than controls, suggesting that bioavailable estradiol may be lower in AD patients [197, 198]. Conversely, one study found that higher estradiol levels were associated with an increased risk of dementia, further emphasizing the complex relationship between estrogen and cognitive health [196].

## 6. Mechanisms of molecular and cellular interactions

### 6.1 Inflammatory factors and the blood-brain barrier

The BBB represents a dynamic combination of physical and chemical boundaries that regulate communication between the CNS and the rest of the body [29]. In a healthy brain, angiogenesis (the growth of new blood vessels from existing ones) helps supply neurons and other brain cells with the oxygen and nutrients required for survival [28]. Angiogenesis works with the tightly regulated blood-brain barrier to maintain brain homeostasis and clear pathological debris [199, 200].

In AD, angiogenesis is impaired [201]. Inflammatory factors can disrupt tight junctions in the BBB, with TNF-α and IL-1β downregulating the expression of tight junction proteins (occludin, claudin-5) and reducing the expression of LRP1 and BCRP proteins, leading to increased BBB permeability [202, 203]. On the other hand, persistent inflammation in the microenvironment can induce apoptosis in endothelial cells, further weakening the integrity of the BBB. This allows peripheral inflammatory factors to enter the brain, exacerbating neuroinflammation in AD [53]. Neuroinflammation, through microglia activation, further intensifies the inflammatory microenvironment and promotes the abnormal phosphorylation of tau protein, leading to neurofibrillary tangles [204]. Additionally, the production of Aβ contributes to BBB disruption and has been found to possess anti-angiogenic properties [205].

Astrocytes play a key role in the construction and maintenance of the BBB. They can express neurotransmitter receptors, respond to external stimuli, and support their development and migration by releasing extracellular matrix (ECM) molecules, such as Tnc. In AD patients, ECM molecules released by reactive astrocytes often exhibit inhibitory effects, which may accelerate neurodegeneration and affect BBB function [206]. Furthermore, microglia are recruited to the brain during the AD process via chemokine 2(CCR2), enhancing the clearance of Aβ [207]. However, the absence of CCR2 reduces microglia and exacerbates Aβ accumulation, highlighting the importance of microglia in maintaining BBB function and clearing neurotoxins [207]. BBB damage forms a vicious cycle, worsening the pathology [207]. Additionally, fibroblast growth factor (FGF) and TGF-β, which impact bone metabolism, may indirectly affect BBB integrity by regulating the function of astrocytes and microglia [10]. Monocyte chemoattractant protein-1 (MCP-1, also known as CCL2) may influence BBB leakage and macrophage polarization, impacting neuronal loss and the progression of neurodegenerative diseases, such as AD [208].

### 6.2 Sharing inflammatory pathways

Many studies suggest these two diseases share similar pathogenic mechanisms [124]. Hormonal imbalances, genetic factors, identical signaling pathways, and neurotransmitter damage have been identified as common mechanisms [209]. The connection between inflammation and these processes remains to be explored further. Chronic low-grade inflammation is common in several chronic diseases, including osteoporosis and AD. In both diseases, long-standing inflammatory responses lead to persistent tissue damage and dysfunction [48]. Various AD mouse models have been shown to exhibit an osteoporotic phenotype [209-214]. Aβ deposition plays a key role in the pathogenesis of AD, particularly in the interaction between neurodegenerative changes and bone-derived factors [48]. Neuroinflammation contributes to the progression of AD [215]. AD shares neuroinflammation-related genes with osteoporosis [178].

Furthermore, AD patients who experience infections show accelerated cognitive decline, which is positively correlated with peripheral TNF levels [216]. There is apathological link between chronic inflammatory diseases, such as periodontitis, and AD. IL-1β and TNF-α are the most important and earliest cytokines upregulated after periodontal infection. Patients with chronic periodontitis are also at higher risk of developing AD [161]. The systemic upregulation of these cytokines promotes a pro-inflammatory environment in the brain, enhancing brain inflammation and worsening the pathology and cognitive deficits in 3×TG-AD mice [162]. Periodontitis may promote cognitive impairment by regulating the Th17/Treg balance inside and outside the brain. The continuous release of these pro-inflammatory cytokines could affect the brain similarly to systemic inflammation [164], potentially linked to the permeability of inflammatory factors through a disrupted blood-brain barrier. Porphyromonas gingivalis, a bacterium associated with periodontitis, secretes gingipains, which could induce systemic inflammation and be related to the pathogenesis of AD. Additionally, the excitotoxic toxin quinolinic acid (QUIN), produced by activated monocytes [161], induces IL-1β expression in astrocytes, thus driving the development of neuroinflammation [165].

The inducible Col1-IL1bXAT osteoarthritis mouse model, characterized by osteoarthritis induced in the knee and temporomandibular joints, activates astrocytes and microglia in the brain, accompanied by upregulation of inflammation-related gene expression. This model explores the biological significance of the link between peripheral inflammation and brain inflammation, showing that osteoarthritis-induced neuroinflammation accelerates and worsens Alzheimer's disease pathology [217].

**Figure 3.**
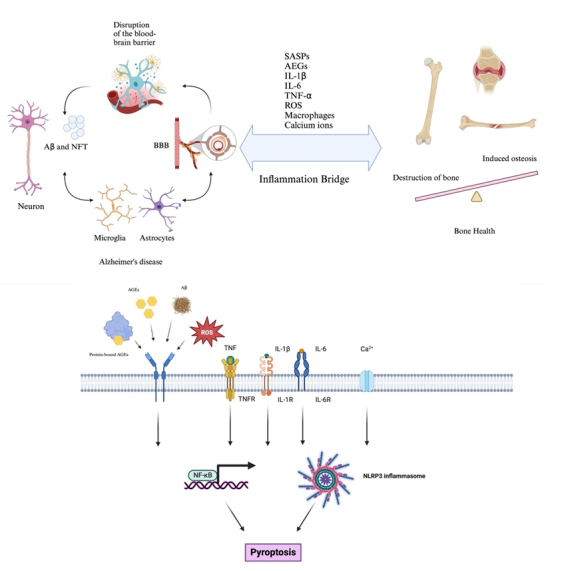
**Pathophysiological Interplay Between Neuroinflammation in Alzheimer’s Disease and Bone Health SASP, AEGs, various inflammatory factors (IL-1β, IL-6, TNF-α), ROS, macrophage infiltration, and calcium ion homeostasis can all serve as an “inflammation bridge” in AD and bone health**. By activating the NLRP3 inflammasome, these factors trigger pyroptosis, ultimately worsening both AD pathology and skeletal integrity. Created by BioRender.

In an analysis of the microglia transcriptome from AD mice to identify common genes involved in osteoclast biology, 35 upregulated genes and 89 downregulated genes were identified. Among these common genes, seven play significant roles in bone homeostasis. Genes such as CSF1, SPP1, FAM20C, and Cst7 were upregulated and are related to osteoclast formation and inflammation, while genes such as LILRA6, MMP9, and COL18A1, which are downregulated, are involved in bone formation and osteoclast regulation [218]. Another study, through intersection analysis of three datasets (GSE97760, GSE168813, and GSE62402), identified 21 common target genes expressed in the peripheral blood samples of AD and osteoporosis patients. Ten core target genes related to these diseases were identified, including CLEC4D, PROK2, SIGLEC7, PDGFB, PTCRA, and ECH1. In the GSE62402 dataset, related to osteoporosis, two differentially expressed mRNAs, Prokineticin 2 (PROK2) and Colony Stimulating Factor 3 (CSF3), were identified [219]. Through network analysis of the skeletal and brain transcriptomes, common molecular mechanisms between the two were found [220]. Analysis of RNA sequencing data from the dorsolateral prefrontal cortex of 629 post-mortem brains and RNA array data from bone biopsies of 84 postmenopausal women revealed co-expressed genes associated with multiple bone and neural features. Pathway enrichment analysis revealed that three modules in the ROSMAP dataset were significantly associated with Alzheimer's disease and bone-related traits. The Wnt pathway was highly enriched in osteoporosis and Alzheimer's disease-related modules [220]. In animal experiments, Wnt/β-catenin signaling genes in htau mice were suppressed, with significant upregulation of extracellular Wnt ligands (Wnt3a) antagonists (Dkk1 and Sost) and significant downregulation of the co-receptor Lrp5, intracellular signaling molecules β-catenin, and the nuclear transcription factor Runx2. Expression levels of bone formation/strength indicators Col1a1 and Col5a1 in htau mice (both male and female) were significantly reduced, indicating that suppression of this signaling pathway might be associated with bone loss in AD [213]. A schematic diagram of the inflammatory bridge is shown in Figure 3.

### 6.2.1 Exosomes and miRNAs

A complex bidirectional communication between the central nervous system and skeletal metabolism [221]. By utilizing mouse models and in vivo fluorescence imaging, researchers have observed that under both normal and pathological conditions, osteocyte-derived extracellular vesicles (OCY-EVs) are transported to the brain, representing a mode of communication between bone and brain [222].

During inflammation, the activation of astrocytes affects the composition of the exosomes they secrete [223]. For instance, in response to inflammatory stimuli, astrocytes release exosomes containing pro-inflammatory factors, which not only modulate local immune responses but may also enhance neuronal vulnerability. The release of exosomes can also be triggered under oxidative stress conditions, and these exosomes can promote the aggregation of Aβ, thereby accelerating the progression of Alzheimer's disease [224]. Exosomes can transport neuroprotective factors, such as antioxidants and neurotrophic factors, aiding in inhibiting neuronal death and promoting neuronal survival. Conversely, activated astrocytes may release exosomes bearing pro-inflammatory cytokines, which could lead to systemic or localized inflammation.

AD-derived brain exosomes (AD-B-EVs) significantly promote the differentiation of bone marrow mesenchymal stem cells (BMSCs) from osteogenesis to adipogenesis, resulting in an imbalance between bone and fat. Additionally, miR-483-5p regulates the anti-osteogenic and pro-adipogenic effects of AD-B-EVs by inhibiting IGF2 [100]. Correspondingly, the elevated levels of OCY-EVs in patients with Alzheimer's are associated with decreased bone density, suggesting that osteocytes secrete exosomes to compensate for the neuronal damage induced by Alzheimer's disease [222]. The increase in miR-483-5p levels negatively correlates with bone density (T-Score), suggesting its potential as a biomarker for assessing AD-related osteoporosis [100]. The combined expression of miR-323-3p and miR-370 in plasma can significantly distinguish patients with MCI from age-matched cognitively normal individuals. This suggests that miR-323-3p may serve as a biomarker for early cognitive impairment, facilitating the early diagnosis of MCI [224].

### 6.2.2 NLRP3

The NLRP3 inflammasome is a multiprotein complex that processes the pro-inflammatory IL-1β and is associated with various inflammatory diseases [225]. Improper activation of NLRP3 can drive the pathogenesis of AD [226]. The NLRP3 inflammasome is an essential regulator of sterile inflammation, activated by host-derived sterile molecules, leading to intracellular activation of caspase-1, processing of pro-inflammatory cytokines IL-1β and IL-18, and pyroptotic cell death. IL-1β contributes to bone resorption. It inhibits osteogenic differentiation by suppressing the BMP/Smad pathway and bone formation markers, including RUNX2, Osteocalcin (OCN), and ALP. IL-1β binds to IL-1R on T cells or B cells, inducing the expression of RANKL on osteoblasts, and then promotes osteoclast activation through a RANKL-RANK-independent mechanism [227].

Intervening with the NLRP3 inhibitor MCC950 in aging-accelerated mice (SAMP8) showed promising results. In vitro experiments demonstrated that pre-treatment with MCC950 in human primary neurons significantly reduced neurotoxicity after Aβ1-42 stimulation. In vivo, MCC950 treatment improved spatial memory and brain tissue morphology in SAMP8 mice and reduced amyloid-beta deposition in the brain. Furthermore, immunofluorescence staining revealed that MCC950 inhibited the overexpression of NLRP3, caspase-1, and GSDMD, which are associated with pyroptosis. These findings suggest that neuron pyroptosis triggered by the NLRP3/caspase-1/GSDMD axis is a crucial factor in neuronal loss in Alzheimer's disease, and MCC950 may be a potential therapeutic agent for AD [228].

### 6.2.2 Oxidative stress

The activation of inflammation through oxidative stress may represent a shared mechanism between AD and bone health, influencing the metabolism of both bone and brain. ROS are essential intracellular redox signaling molecules regulating bone metabolism. Their generation affects cellular stress response mechanisms and is regulated by genes encoded by extended sequences [118].

Oxidative damage is related to impaired glucose and glutamate transport and mitochondrial dysfunction induced by Aβ in synaptosomes [229]. Oxidative damage, particularly leading to mitochondrial dysfunction and abnormal energy metabolism, may also interfere with the glutamate-glutamine cycle, causing an increase in neuronal calcium ion levels, which in turn triggers excitotoxicity and apoptosis [230]. Additionally, dysfunction in the interactions between neurons and glial cells can lead to chronic neuroinflammation, which promotes long-term microglial activation, ultimately resulting in neurodegeneration and functional decline [63].

Furthermore, the oxidation of glycated proteins leads to the accumulation of advanced glycation end products (AGEs) in the extracellular space. AGEs are generated by the non-enzymatic modification of macromolecules (such as proteins, lipids, and nucleic acids) by sugars (e.g., glucose, fructose, and pentose) through the Maillard reaction. AGE molecules can be metabolized and cleared in the proximal tubular cells of the kidney through aldose reductase I and II [231]. AGEs, such as carboxymethyl lysine (CML) and pentosidine (PEN), have been identified in bone tissue, and their accumulation is associated with the deterioration of bone quality [232]. The increase of AGEs is not only observed in bone tissue but is also linked to oxidative stress and chronic inflammation, which may further contribute to the progression of osteoporosis. AGEs induce inflammation and immune suppression by binding to the AGE receptor (RAGE), altering innate and adaptive immune responses, generating pro-inflammatory cytokines, ROS, and reactive nitrogen intermediates (RNI). This represents another mechanism by which RAGE leads to neuronal degeneration in AD [233]. In the 5XFAD transgenic mouse model, the accumulation of Aβ42 in the brain was associated with increased AGEs in bone. In Tg2576 mice expressing the Swedish mutation, a biphasic effect on osteoclast (OC) activation was observed: young mice (<4 months) showed increased OC numbers.In comparison, older Tg2576 mice (>4 months) exhibited reduced OC numbers. The increase in OC numbers in young Tg2576 mice seemed to be mediated by the expression of Aβ oligomers and RAGE in bone marrow macrophages. However, the reduced OC formation and activity in older Tg2576 mice may be due to increased soluble RAGE (sRAGE), an inhibitor of RANKL-induced osteoclastogenesis. These findings suggest that APPswe/Aβ has unexpected functions and reveals mechanisms underlying altered bone remodeling in AD patients, identifying APP/Aβ and RAGE as common factors linking AD and osteoporosis [234].

Aging is a primary risk factor for neurodegenerative diseases. During aging, microglial cells undergo phenotypic changes that may lead to neuroinflammation, which is associated with the pathogenesis of diseases such as AD. Aging results in the abnormal activation of microglial cells, characterized by increased pro-inflammatory cytokines and excessive production of ROS. This activation is closely linked to cognitive impairment and neurodegeneration. The mitochondrial function of microglial cells is compromised during aging, leading to increased ROS production, further exacerbating neuroinflammation and creating a vicious cycle [63]. The specific mechanism involves APPswe in osteoblasts, rather than APPwt or APPlon (the London variant), which triggers endoplasmic reticulum stress-driven senescence, resulting in systemic and cortical inflammation as well as behavioral changes in 6-month-old TgAPPsweOCN mice [211]. The migratory capacity, phagocytic ability, and microglia responsiveness to injury diminish with advancing age. This functional decline results in a reduced capacity to clear Aβ. Aging induces alterations in the brain microenvironment, including establishing a chronic low-grade inflammatory state. Such an environment may predispose microglia to maintain a pro-inflammatory state, inhibiting their normal phagocytic and clearance functions, including removing Aβ. The elevated levels of aging-associated pro-inflammatory cytokines exacerbate inflammation rather than exerting a regulatory effect. The expression level of the receptor CD36, which interacts with Aβ on microglia, may decrease with age. This reduction in expression could impair the ability of microglia to recognize and clear Aβ, leading to its accumulation. Aging is also accompanied by increased oxidative stress, which may compromise the cellular functions of microglia. Oxidative stress can affect the signaling pathways of microglia, potentially resulting in an inadequate or insufficiently robust response to Aβ [235].

The RANK/RANKL/OPG signaling pathway plays a role in bone metabolism. It regulates inflammatory responses within the nervous system, which may influence their interplay. Microglial cells can promote bone resorption and affect skeletal health by secreting RANKL. Patients with AD often exhibit elevated levels of ROS, which can lead to neuronal damage and death, while also impacting bone metabolism. ROS not only facilitates bone resorption but also inhibits the differentiation of osteoblasts, resulting in a decline in bone quality. Neuroinflammation can lead to alterations in the levels of collagen associated with osteogenesis, such as COL1A1 and COL1A2, thereby directly impacting osteoporosis [177]. Under the stimulation of external oxidative stress, Nrf2 is activated and translocates to the nucleus. This process initiates the transcription of downstream antioxidant proteins, thereby protecting cells from oxidative damage. However, Nrf2 is primarily localized in the cytoplasm of hippocampal neurons, and the nuclear protein levels are significantly reduced in the Alzheimer's disease microenvironment. Defects in the Nrf2-mediated signaling pathway can lead to the accumulation of ROS and neuronal injury [236].

In addition to oxidative stress effects, estrogen withdrawal associated with menopause may make bones more susceptible to oxidative damage, thereby increasing the risk of postmenopausal osteoporosis [232]. Systemic Wnt/β-catenin signaling defects can disrupt bone homeostasis [40, 237]. Furthermore, ROS stimulate RANKL activation through ERK and NF-κB, leading to osteoclastogenesis. These factors are associated with bone homeostasis imbalance, favoring bone resorption over bone formation [238].

### 6.2.3 Macrophages

In the pathological changes of osteoporosis and AD, macrophage populations in bone and brain (osteoblasts and microglial cells) are responsible for the disease progression [239]. Macrophages, microglia, and osteoclasts originate from the mononuclear/macrophage lineage. These cells arise from embryonic blood islands and undergo a series of developmental stages, ultimately maturing into specific tissue-resident cells. During this developmental process, they experience similar pathways and mechanisms of origin, resulting in similarities in specific biological characteristics. The signaling complex formed by TREM2 and DAP12 is expressed in both macrophages and microglia, playing a crucial role in the functions of these two cell types. In AD, the functional deficiency of TREM2 is closely associated with the accumulation of Aβ plaques, subsequently affecting microglia's phagocytic and clearance capabilities [239]. In microglia, the activation of TREM2 facilitates the phagocytosis of Aβ, thereby clearing amyloid plaques and preventing neuronal damage. The formation of osteoclasts relies on the synergistic action of CSF1 and RANKL, both promoting osteoclast precursors' differentiation and function [239].

In the early stages of AD, Aβ can transiently induce the nuclear translocation of TFEB; however, over time, the decline in nuclear levels of TFEB and the sustained increase in LAMP1 (lysosomal-associated membrane protein 1) indicate that the cellular adaptive response to Aβ is limited, ultimately leading to lysosomal dysfunction. TFEB is a critical transcription factor responsible for regulating the biosynthesis and function of lysosomes. When TFEB fails to translocate into the nucleus, it cannot exert its role in promoting lysosomal function, thereby inhibiting the phagocytic capacity of microglia [71]. The pleiotropic phenomenon (i.e., genetic variations influencing multiple traits) suggests that the Pyk2-mediated actin polymerization pathway in osteoblasts and microglial cells may be a pleiotropic mediator linking osteoporosis and AD as shared risk factors [239]. Furthermore, the polarization characteristics of macrophages and the differences in the paracrine factors they secrete influence the development of postmenopausal osteoporosis [103].

It has been found that the clearance of Aβ protein especially requires the expression of CCR2 in bone marrow cells (BMCs) [46]. In APP(Swe)/PS1 mice (transgenic mice expressing chimeric amyloid precursor protein and human PS1), disease progression is aggravated in BMCs lacking CCR2. Transplantation of CCR2-deficient BMCs further exacerbates memory deficits, increases the levels of soluble Aβ, and enhances the expression of transforming growth factor (TGF)-β1 and its receptor. In contrast, wild-type bone marrow stem cell transplantation restores memory function and reduces soluble Aβ accumulation in APP(Swe)/PS1 and APP(Swe)/PS1/CCR2^-^/^-^ mice. Finally, gene therapy with lentivirus carrying CCR2 in BMCs can prevent cognitive decline in AD model mice. The reduced expression of CCR2 in bone marrow-derived microglia may play a crucial role in the pathogenesis of this neurodegenerative disease [46].

Macrophages participate in neuroinflammation or pain development through the activation of cyclooxygenase-2 (COX-2), which leads to the generation of prostaglandin E2 (PGE2) [240]. Bone marrow-derived macrophages can effectively reduce the deposition of Aβ in the brain [17, 46, 241]. Macrophages from wild-type mice can degrade soluble and insoluble Aβ time-dependently and significantly eliminate amyloid deposits. Macrophages expressing ApoE2 degrade Aβ more efficiently than macrophages expressing ApoE3, ApoE4, or lacking ApoE [17]. Moreover, anti-ApoE antibodies effectively block macrophage-mediated Aβ degradation. Macrophages expressing ApoE2 show higher matrix metalloproteinase-9 (MMP-9) activity, which is upregulated by various pro-inflammatory mediators in CNS diseases [242]. Bradykinin (BK), a common pro-inflammatory mediator, is elevated in multiple brain injuries and inflammatory diseases. Elevated BK may adversely affect the CNS by upregulating MMP-9 or promoting COX-2-derived PGE2 generation in astrocytes, exacerbating brain inflammation. A novel autocrine pathway of BK-induced MMP-9 protein expression is mediated by the activation of STAT3, relying on endogenous COX-2/PGE2-mediated c-Src/Jak2/ERK signaling in astrocytes [242].

IL-17 induces a decrease in bone density by promoting the differentiation and activity of osteoclasts. Specifically, it can stimulate osteoblasts to secrete RANKL. IL-17 relies on glutamine (Glu) metabolism to support the energy demands of osteoclasts, thereby facilitating their differentiation and function [243]. IL-34-activated macrophages show significantly reduced uptake of Aβ42, with distinct differences in immunophenotype and activation phenotype compared to CSF1-cultured macrophages [241]. Additionally, macrophages treated with IL-34 express lower levels of related proteins.Furthermore, IL-34-treated monocytes differentiate into fewer mature macrophages compared to CSF1-treated monocytes. These findings suggest that IL-34 may impair monocytes' ability to differentiate into macrophages and reduce their ability to uptake pathological Aβ42 [241]. Given the vital role of macrophage-mediated Aβ clearance in AD models and patients, future research should consider regulating IL-34 to enhance macrophage-mediated Aβ clearance and prevent disease progression.

The degradation of Aβ typically relies on the formation and functionality of autophagosomes, which facilitate the transport of these proteins to lysosomes for degradation. Additionally, autophagy further regulates the expression of genes associated with Aβ production by influencing transcription factors such as SP1, CTCF, and NF-κB [244]. OPTN is an autophagy receptor that facilitates the clearance of autophagy-related proteins and organelles [210]. It enhances the process of selective autophagy by binding with relevant autophagosomes, aiding in the removal of harmful intracellular substances, including Aβ. OPTN can promote the proliferation and osteogenesis of BMSCs.

Additionally, targeting autophagy receptors such as Optineurin (OPTN) and Sequestosome 1 (SQSTM1) can increase bone density and strength in AD mice, promote Aβ clearance, and maintain bone homeostasis [210]. Aβ1-42 directly inhibits the proliferation of BMSCs, while the AKT/mTOR signaling pathway plays a crucial role in regulating autophagy levels. Furthermore, autophagy may serve as a resistance mechanism against the reduction in BMSC proliferation induced by Aβ1-42 [245].

### 6.2.4 Calcium imbalance

Aβ oligomers or HypF-N oligomers can directly interact with the cell membrane, impairing the membrane's structure and function. This interaction may increase membrane permeability, disrupting the Ca^2+^ balance between the intracellular and extracellular environments, thereby causing abnormal Ca^2+^ influx. When aggregates bind to the lipid bilayer, they may form tiny pores within the membrane, increasing its permeability to Ca^2+^ and dramatically increasing intracellular Ca^2+^ concentration. Once Aβ oligomers associate with the cell membrane, they may induce Ca^2+^ entry through interactions with NMDA and AMPA receptors. This non-specific membrane disruption mechanism could significantly contribute to calcium overload [51]. As the function of astrocytes gradually declines, it may lead to calcium loss in the bones, triggering osteoporosis. This condition results in an excessive increase in calcium ions in the bloodstream, which can subsequently deposit in the pineal gland, causing ectopic calcification and promoting the development of AD [246]. Therefore, it has been hypothesized that astrocyte dysfunction may lead to prolonged calcium imbalance in the body, thereby contributing to the co-occurrence of osteoporosis and AD [63, 230]. Osteocytes are also a type of astrocyte, and when the function of astrocytes gradually becomes dysregulated, it may lead to the loss of calcium ions from the bones, thereby triggering osteoporosis. When calcium ions are released from the skeletal system into the bloodstream, calcium ions may accumulate within the body, ultimately depositing in the pineal gland, leading to calcification [230].

In the brains of AD model mice and patients, the expression of calcium-activated potassium channel KCa3.1 is significantly increased [247]. Blocking KCa3.1 with pharmacological agents can substantially reduce astrocyte proliferation and microglial activation, alleviating neuronal loss and improving memory deficits. In AD mouse models, the expression of calcium homeostasis modulator 2 (Calhm2) is increased. Knocking out Calhm2 in 5×FAD mice, which carry five familial AD gene mutations, significantly reduces amyloid β deposition and neuroinflammation and improves cognitive deficits [247].

Disruption of mitochondrial calcium homeostasis is also linked to tau protein and other risk factors in AD. However, a persistent challenge in this field is the inconsistency of data collected from different models or experimental environments [248]. Calcium-sensing receptor (CaSR), a member of the class C G-protein-coupled receptors (GPCRs), plays a critical role in calcium homeostasis [249]. The discovery that soluble Aβ (sAβ) can bind to CaSR and act as a CaSR agonist suggests a key mechanistic role. The sAβ-CaSR interaction promotes the overexpression of new Aβ oligomers, which progressively accumulate in neurons and cause subsequent cytotoxic effects. This effect can be mitigated by downregulating CaSR or using CaSR inhibitors [250]. CaSR directly regulates the expression of pro-inflammatory cytokines in neurons [251]. As a hallmark of AD, Aβ accumulates in osteoporotic bone samples and activates osteoclasts, thereby contributing to bone loss. Compared to age- or sex-matched normal bone, the expression levels of Aβ42 and APP are significantly elevated in the skeletal tissues of osteoporosis patients and ovariectomized rats, and the expression levels of both are negatively correlated with the bone density of the patients [252]. The specific mechanism enhances RANKL-induced osteoclast activation through the signaling pathways of NF-κB, ERK, and calcium oscillations [50].

## 7. Therapeutic implications and interventions

### 7.1 Current therapeutic approaches for AD inflammation

Patients with AD typically exhibit cognitive decline, memory loss, as well as impairments in synaptic plasticity and energy metabolism. Current pharmacological interventions for AD primarily alleviate symptoms without addressing the underlying pathological mechanisms [97]. Commonly utilized medications include acetylcholinesterase inhibitors, such as donepezil, and NMDA receptor antagonists, such as memantine; however, these agents do not reverse or halt disease progression and are classified as symptomatic treatments [253]. The use of non-steroidal anti-inflammatory drugs (NSAIDs) in the treatment of AD has been noted, although these medications have demonstrated limited efficacy in clinical trials [254]. Small molecule inhibitors targeting TNF-α and IL-1β have entered clinical trial phases, yet some studies have failed to progress due to insufficient therapeutic effects [255]. Aβ is one of the core mechanisms underlying AD, and extensive research has been conducted on Aβ clearance [236]. Trials involving three distinct Aβ antibodies (Solanezumab, crenezumab, and aducanumab) indicate a deceleration in cognitive decline in post-hoc analyses of subjects with mild AD [256]. Targeted antibody therapies against Aβ that have received approval from the U.S. FDA include Lecanemab and Aducanumab, which are associated with significant side effects [257]. Aducanumab has demonstrated effects in delaying clinical deterioration across multiple studies; however, it is accompanied by an elevated risk of amyloid-related imaging abnormalities—edema (ARIA-E), necessitating careful management in clinical practice [258, 259]. Targeting soluble and insoluble Aβ peptides for degradation has significantly reduced Aβ plaques and conferred clinically meaningful cognitive benefits [260], while potentially exerting indirect effects in suppressing neuroinflammation [253].

### 7.2 Current therapeutic approaches for inflammation related to skeletal health

Currently, the treatment of osteoporosis-related inflammation primarily focuses on reducing bone destruction mediated by inflammatory factors and promoting bone regeneration. Osteoporosis treatment modalities include bisphosphonates, monoclonal antibodies, parathyroid hormone (PTH), and abaloparatide. Bisphosphonate medications, such as alendronate and risedronate, can inhibit bone resorption, thereby maintaining bone density [249]. Additionally, axial spondyloarthritis (axSpA) is associated with an increased risk of osteoporosis prevalence. Alendronate is recommended for managing low BMD in patients with axSpA; however, evidence does not support the use of TNF inhibitors for treating low BMD [260].

On one hand, using NSAIDs or moderate doses of glucocorticoids can alleviate inflammatory responses within bone tissue in the short term; however, the long-term or high-dose use of glucocorticoids may accelerate bone loss, necessitating careful consideration. Licofelone is a drug approved for the treatment of osteoarthritis that simultaneously inhibits the cyclooxygenase (COX) and lipoxygenase (5-LOX) pathways, exhibiting analgesic and anti-inflammatory effects. Additionally, it has demonstrated neuroprotective properties within the central nervous system, which are associated with its regulatory effects on the COX/5-LOX pathways, inflammatory cytokines, and immune responses [261]. On the other hand, biologics targeting specific inflammatory factors, such as TNF or IL monoclonal antibodies, have also shown potential value in clinical studies, as they can inhibit bone resorption and improve bone remodeling balance [262].

**Figure 4.**
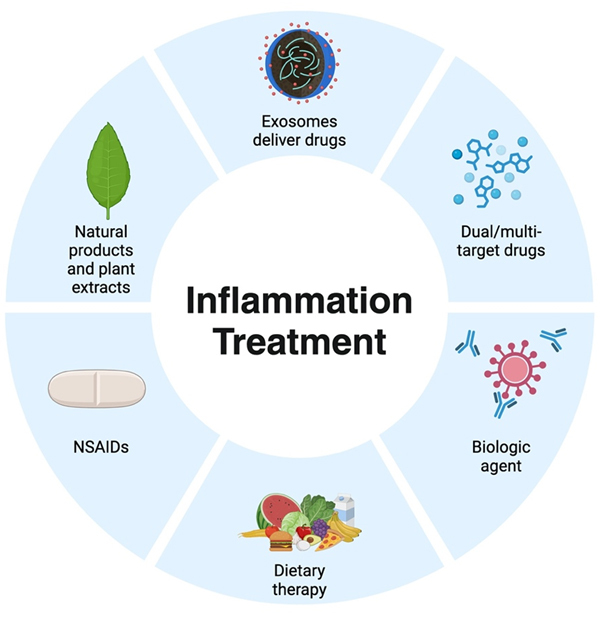
**Inflammation Treatment**. Encompassing natural products and plant extracts with their anti-inflammatory and antioxidant activities, non-steroidal anti-inflammatory drugs (NSAIDs) that inhibit prostaglandin synthesis, and dietary therapies or anti-inflammatory diet to regulate immune responses. Biologic agents, including targeted antibodies and cytokine-based approaches, selectively block inflammatory mediators, while dual- or multi-target drugs address multiple signaling pathways simultaneously to bolster efficacy and minimize resistance. Exosome-based delivery systems further enhance treatment precision by transporting medications directly to inflammatory sites, thereby reducing systemic toxicity and improving therapeutic outcomes. Created by BioRender.

Odanacatib is a selective cathepsin K inhibitor that represents an emerging approach to inhibit osteoclast-mediated bone resorption, potentially aiding in the separation of bone formation from bone resorption [263]. The monoclonal antibody romosozumab has been shown to increase bone mineral density in postmenopausal women and reduce the incidence of vertebral fractures by binding and inactivating sclerostin, a glycoprotein secreted by osteocytes, thereby inhibiting osteoblast proliferation through the blockade of the Wnt signaling pathway [264]. Denosumab is a monoclonal antibody that inhibits the development and activity of osteoclasts by blocking RANKL, thereby preserving BMD [265]. Denosumab is a fully human monoclonal antibody targeting the receptor activator of RANKL, which has demonstrated efficacy in preventing vertebral, non-vertebral, and hip fractures. Its administration via subcutaneous injection every six months may significantly impact patient adherence and treatment persistence [266].

### 7.3 Potential Cross-Benefits of Therapeutic Interventions

Future medical strategies should adopt a comprehensive, patient-centered approach that focuses on cognitive function and emphasizes skeletal health, enhancing the overall health status of patients affected by both conditions. This approach integrates clinical practice with the challenges and opportunities faced by elderly individuals with cognitive impairment or dementia, particularly in the prevention of high-risk fragility fractures [267]. Such strategies improve patient health and survival rates and reduce healthcare costs and socioeconomic burdens [127]. Multimodal physical exercise programs have decreased the risk of falls and improved gait, balance, and bone density, particularly demonstrating significant effects in short- and medium-term AD patients [268]. Interdisciplinary interventions, including physical therapy, occupational therapy, cognitive training, and environmental adaptations, can enhance patients' quality of life, delay cognitive decline, and improve their ability to perform activities of daily living [134].

## 8. Future research directions

In recent years, clinical trials of anti-inflammatory drugs for the treatment of AD have not achieved significant success [254]. Nevertheless, many candidate compounds are still regarded as potential therapeutic agents, particularly cocktail therapies that combine multiple treatment modalities, which may yield breakthroughs in future research [269]. Unlike traditional anti-inflammatory strategies that inhibit the synthesis of inflammatory mediators, novel inflammation resolution strategies aim to restore the normal physiological processes of inflammation and terminate abnormal inflammatory responses [270]. Due to AD's biological, genetic, and clinical complexities, the development of existing therapeutic agents faces substantial challenges. Therefore, there is an urgent need to integrate research programs for novel drug discovery, especially those based on reliable biomarkers, including clinical phenotypes, genetic backgrounds, and neuroimaging data, to guide treatment [271]. In summary, there are numerous gaps in the current treatment of AD, particularly in the integrative research of anti-inflammatory therapies and skeletal health, necessitating further exploration and integrating new therapeutic approaches. As shown in Figure 4.

### 8.1 Novel Drugs

Novel therapeutic drugs are shown in Table 1. Aβ clearance represents a novel therapeutic agent with significant potential, as evidenced by Donanemab's substantial reduction of the plasma biomarkers PTAU217 and neurofilament light chain over a 72-week treatment period, indicating its potential impact on AD pathology. Similarly, BIIB080 has demonstrated effects on TAU biomarkers and cognitive decline in Phase II trials and is currently under further evaluation [272, 273].

**Table 1:** Recent clinical research studies.

Interventions	Mechanism/Hypothesis	Trial Phase/Study Type	Sample population	Time	Outcome	Prospect	Ref.
**Leptin and IGF-1**	An investigation was conducted to explore how leptin and IGF-1 mediate the relationship between hypothalamic atrophy and bone loss.	Longitudinal Study	Seventy-one older adults with early AD and 69 non-dementia controls were two-year observational studies	Two years	No significant indirect effects of leptin or IGF-1 were found	The relationship between AD and bone loss can be complex and multiple, and a simple causal model may not adequately explain this relationship.	[124]
**MAPTRx**	Safety and Targeted Effect of MAPTRx, an Antisense Oligonucleotide Against Tau Protein, in Patients With Mild AD.	Phase IB	Forty-six patients, 34 were randomised to MAPTRX and 12 were given placebo.	23 weeks	The concentration of total Tau protein in CSF in the MAPTRx-treated group showed a dose-dependent reduction. In patients receiving higher doses (such as 60 mg and 115 mg), a reduction in T-Tau was observed by about 50% within 24 weeks after treatment. This reduction persisted for 24 weeks after the end of treatment, suggesting a lasting effect of the therapy on T-Tau.	Despite a decline in the clinical manifestations of participants, which aligns with the expected disease progression of mild Alzheimer's disease, MAPTRx has demonstrated potential therapeutic effects on pathological biomarkers, warranting further evaluation in larger-scale clinical trials.	[278]
**Aerobic cycling**	Evaluate the impact of a 6-month aerobic cycling intervention on cognitive function in patients with AD.	Randomized Controlled Trial	A total of 96 participants, of whom 55% were male, had an average age of 77.4 years, and the average years of education among the participants was 15.6 years.	6 months	After a six-month intervention, there were no significant intergroup differences in the cognitive domains (attention, executive function, and language) between the two groups.	Although the study did not find significant intergroup differences, aerobic exercise may have a positive impact on the quality of life of patients, and it is associated with fewer side effects.	[367]
**Brexpiprazole**	Brexpiprazole is a novel atypical antipsychotic medication developed by the biopharmaceutical companies Otsuka Pharmaceutical and H. Lundbeck. It acts as a partial agonist at the 5-HT1A receptor while exhibiting antagonistic effects on receptors such as 5-HT2A, D2, α1B, and α2C.	Randomized Controlled Trial	Study 1 (fixed dose): 433 participants randomized; Study 2 (flexible dose): 270 participants randomly allocated.	Both studies lasted for 12 weeks.	Brexpiprazole 2 mg/day may represent an effective, safe, and well-tolerated therapeutic option for the treatment of agitation symptoms in dementia among the elderly, addressing the current demand for pharmacological interventions in this field.	Brexpiprazole also demonstrated a greater improvement than placebo in the Clinical Global Impression-Severity (CGI-S) scores, although it did not achieve statistical significance in certain comparisons.	[280]
**Brexpiprazole**	The same as above.	Randomized Controlled Trial	A total of 345 patients were recruited, of which 228 received brexpiprazole and 117 received a placebo.	12 weeks	Patients who received brexpiprazole (2 or 3 mg) had significantly better improvement on the Cohen-Mansfield Agonistic Scale (CMAI) total score than in the placebo group.	It is recommended that more in-depth research be conducted in the future.	[279]
**Brexprazole**	A novel antipsychotic medication, classified as an atypical antipsychotic.	Phase II/III	A total of 410 patients were eligible and randomly assigned to groups: 112 patients received brexpiprazole at 1 mg, 149 patients received brexpiprazole at 2 mg, and 149 patients received a placebo.	10 weeks	The efficacy of Brexprazole at a dosage of 1 mg per day has been confirmed for the first time.	The research plan involves a long-term (24-week) extended study to further evaluate the safety and efficacy of Brexpiprazole in patients with AAD.	[281]
**Plasma exchange**	It is hypothesized that albumin and intravenous immunoglobulin (IVIG) replacement is effective in the treatment of AD	Phase IIB/III	A total of 347 participants were included.	12 months	Plasma exchange combined with albumin replacement therapy may slow cognitive and functional decline in patients with AD, demonstrating safety and feasibility.	It is recommended that more in-depth research be conducted in the future.	[287]
**Fortasyn Connect**	A specific combination of multi-nutrients (Fortasyn Connect) is effective for cognitive function and related indicators.	Randomized Controlled Trial	A total of 311 participants were recruited.	36 months	The specific multi-nutrient combination Fortasyn Connect demonstrated a significant improvement in cognitive function and a reduction in brain atrophy after a 36-month intervention in patients with prodromal Alzheimer's disease, as evidenced by a notable decrease of 45% in the Clinical Dementia Rating Scale-Sum of Boxes (CDR-SB).	Future research could explore methods for multimodal interventions at earlier stages and over extended durations to further enhance potential therapeutic effects.	[310]
**Posiphen**	A small molecular compound that can reduce the translation of APP by binding to the iron-responsive element (IRE) within the APP mRNA.	Phase IB Randomized Dose Escalation Clinical Trial	A total of 19 participants were recruited and randomly assigned within the dosage groups (5 active group participants receiving 60 mg once daily and 5 active group participants receiving 60 mg twice daily, along with 3 participants in the placebo group).	23 days	The synthesis rate of CSF Aβ40 (FSR) did not exhibit significant overall or dose-dependent effects between the Posiphen group and the placebo group. Comprehensive multi-parameter modeling supports a dose-dependent reduction of APP production by Posiphen. There were no significant changes in cognitive measures and CSF biomarkers between baseline and 21 days in the Posiphen group compared to the placebo group.	Although the sample size of this study is relatively small and has not sufficiently demonstrated a significant improvement in cognitive function due to Posiphen, the safety and tolerability data provide support for future clinical trials in patients with early Alzheimer's disease.	[275]
**Aducanumab**	Aducanumab selectively targets aggregated forms of Aβ.	Phase IB	196 patients	48 months	The clinical decline scores in the fixed-dose group of 10 mg/kg and the gradually increasing dose group were significantly lower than those in the placebo switch group.	ARIA-E are the most common adverse events. Dose adjustments are associated with a reduction in the incidence of ARIA-E.	[259]
**CT1812**	CT1812 is a novel brain-penetrant sigma-2 receptor ligand that can interfere with the binding of Aβ oligomers to neurons.	Phase I/II	23 participants with mild to moderate AD dementia	24 weeks	There were no significant differences between the CT1812 group and the placebo group in terms of SV2A or FDG PET signal changes, cognitive clinical scores, or CSF biomarkers. Composite regional volume MRI indicated a notable trend towards tissue protection in the CT1812 treatment group, with nominally significant differences observed in both dosage groups compared to the placebo in the anterior cingulate, prefrontal cortex, and hippocampal cortex.	The 24-week safety results provided by this study, along with the observed changes in volumetric MRI associated with CT1812, support its further clinical development.	[283]
**NeuroEPO plus**	A recombinant human erythropoietin, which lacks erythropoietic activity due to its low sialic acid content, exhibits a shorter plasma half-life. This drug has the potential to prevent oxidative damage, neuroinflammation, apoptosis, and cognitive deficits in models of AD.	Phase II/III	A total of 174 participants aged 50 years and older were diagnosed with mild to moderate Alzheimer's disease clinical syndrome.	48 weeks	In participants with mild to moderate clinical symptoms of AD, NeuroEPO plus improved cognitive assessment over a 48-week period, demonstrating excellent safety.	Larger-scale trials are needed to further ascertain the efficacy and safety of NeuroEPO plus in Alzheimer's disease.	[282]
**Tilavonemab**	Anti-tau monoclonal antibodies	Phase II	453 patients	96 weeks	The efficacy of tilavonemab in treating patients with early Alzheimer's disease has not been demonstrated, despite its target binding showing effectiveness.	It is essential to gain an in-depth understanding of the impact on the pathogenic mechanisms of Alzheimer's disease, as it may involve multiple factors such as amyloid proteins, tau proteins, and neuroinflammation. Treatment targeting tau alone may be insufficient to address the progression of chronic lesions.	[277]
**Aducanumab**	Aducanumab selectively targets aggregated forms of Aβ.	Two Phase III clinical trials	The occurrence of amy-related imaging abnormalities (ARIA) among 3,285 participants.		Among the patients receiving aducanumab treatment, a significant proportion experienced ARIA. Specifically, in the 10 mg/kg dosage group, 425 participants (41.3%) exhibited ARIA, while 362 patients (35.2%) experienced ARIA-edema (ARIA-E). In contrast, only 29 participants (2.7%) in the placebo group exhibited ARIA-E.	It can assist physicians in better monitoring and managing ARIA in early Alzheimer's disease patients receiving Aducanumab in real-world settings.	[258]
**Masitinib**	Masitinib is an oral tyrosine kinase inhibitor that targets activated cells of the neuroimmune system, specifically mast cells and microglia.	A randomized, double-blind, parallel-group (four groups) placebo-controlled trial	718 patients	12 weeks	Masitinib (4.5 mg/kg/day) may be beneficial for patients with mild to moderate AD. A confirmatory study has been initiated to validate these findings.	Initiate confirmatory research to validate these data.	[286]
**GV-971**	A type of oligosaccharide derived from the ocean, exhibiting a novel mechanism of action.	Phase III	818 participants	36 weeks	GV-971 has demonstrated significant efficacy in improving cognition, with sustained improvement observed throughout the 36-week observation period.	There are controversies with unclear mechanisms that require further clinical research.	[368]
**Intranasal Insulin Therapy**	The use of insulin in the treatment of mild cognitive impairment and AD dementia shows considerable potential.	Phase II/III	432 participants	12 months	In the 12-month treatment period, intranasal insulin did not demonstrate cognitive or functional benefits in the primary intention-to-treat population.	Despite the theoretical potential of intranasal insulin, the anticipated clinical improvement was not achieved in this trial.	[369]
**Semorinemab**	Anti-tau monoclonal antibodies	Randomized, placebo-controlled, double-blind	624 participants	Lasting for 48 or 60 weeks, with a maximum duration of up to 96 weeks.	Semorinemab failed to mitigate functional decline in patients with moderate to mild Alzheimer's disease.	Promote further exploration of semorinemab or other anti-tau therapies in moderate to mild AD.	[276]
**Donanemab**	Analyze the association between Donanemab treatment and plasma biomarkers related to AD.	Randomized, double-blind, placebo-controlled clinical trial.	272 participants	Lasts 72 weeks	Significant reduction of plasma biomarkers PTAU217 and neurofilament light chain protein.	These readily accessible plasma biomarkers may provide additional evidence of pathological changes associated with Alzheimer's disease through anti-amyloid therapy. The utility of assessing treatment response will require further evaluation.	[273]
**BIIB080**	Analyze the association between Donanemab treatment and plasma biomarkers, such as pTau217 and glial fibrillary acidic protein (GFAP), to explore its potential therapeutic effects.	Randomized, double-blind, placebo-controlled clinical trial.	Among the 102 participants assessed for eligibility, 46 participants with mild AD were admitted.	36 weeks	BIIB080 reduced TAU biomarkers, including CSF T-TAU, CSF P-TAU181, and TAU PET, which are associated with cognitive decline, a hallmark related to diminished cognitive function.	The impact of BIIB080 on biomarkers and clinical outcomes is being further evaluated in a phase II trial.	[272]
**Intracerebroventricular injection of human umbilical cord blood-derived mesenchymal stem cells (hUCB-MSCs)**	In animal studies, the transplantation of mesenchymal stem cells (MS) has been associated with favorable functional outcomes and a reduction in amyloid protein levels.	Phase I	Nine patients with mild to moderate AD dementia.	Conduct an additional 36 months in the extended observational study.	Feasible, relatively safe, and exhibiting good tolerance.	An extended follow-up study is being conducted for individuals participating in the IIa clinical trial.	[288]
**Lomecel-B**	Assuming that Lomecel-B is an allogeneic drug candidate for the treatment of AD, it is a safe and potentially disease-modifying therapy that operates through a multifaceted mechanism of action.	Phase I	33 patients with mild AD	Serious adverse events (SAE) occurring within 30 days post-treatment.	The results of this trial support the safety of Lomecel-B and demonstrate potential efficacy in preliminary exploratory assessments, providing data support for subsequent larger-scale clinical trials.	Conduct Phase II trials to further validate the efficacy and mechanisms of Lomecel-B, particularly focusing on dose-dependent effects, pharmacokinetics, and the potential benefits of repeated administration.	[289]
**Gamma transcranial alternating current stimulation (tACS)**	Elucidate the beneficial effects of gamma tACS on cognitive functions in Alzheimer's disease and investigate its impact on gamma oscillatory activity in the hippocampal region.	A double-blind, randomized controlled single-center trial.	46 patients	Received 30 sessions of one-hour 40Hz tACS or placebo stimulation over a continuous period of 15 days.	The enhancement effects of θ-gamma coupling and neural activity within the hippocampus suggest a potential mechanistic model for the therapeutic effects of tACS.	Continue to propose potential mechanisms.	[290]
**Docosahexaenoic acid (DHA) supplements**	It is assumed that a higher dosage of DHA is required to ensure adequate bioavailability in the brain, and that the APOE4 genotype is associated with a reduced delivery of DHA and eicosapentaenoic acid (EPA) to the brain prior to the onset of cognitive impairment.	Randomized placebo-controlled clinical trial	33 participants	6 months	Dementia prevention trials utilizing omega-3 supplements at daily doses equal to or less than 1 gram may have minimal effects on the brain, particularly among APOE4 carriers.	The conclusion of this clinical trial emphasizes the potential for further research on high doses of DHA, particularly in evaluating its effects on cognitive function in patients with Alzheimer's disease.	[308]
**Methylphenidate**	A central nervous system stimulant, widely used for the treatment of Attention Deficit Hyperactivity Disorder (ADHD) and Narcolepsy, may also be utilized in the treatment of AD.	Multicenter Randomized Placebo-Controlled Clinical Trial	307 participants	6 months	Methylphenidate is a safe and effective medication that can be used to treat apathy in AD.	It is recommended to conduct larger-scale and long-term clinical studies.	[285]
**Neprilysin**	Neprilysin is physiologically responsible for the clearance of Aβ in the body. Sacubitril/valsartan is a commonly used neprilysin inhibitor, which is a combination medication.	Randomized clinical trials	92 patients	52 weeks	Sacubitril/valsartan may have a confounding effect on blood biomarkers for Alzheimer's disease, particularly leading to misinterpretation when assessing the Aβ42/Aβ40 ratio.	In clinical practice, it is essential to interpret changes in relevant biomarkers with caution.	[274]
**High-dose vitamin D followed by intranasal insulin.**	Nasal insulin can rapidly enhance cognitive function, while vitamin D can upregulate insulin receptor expression, thereby augmenting insulin action.	Randomized clinical trials	63 patients	8 weeks	High-dose vitamin D does not provide additional benefits for cognition or disability in individuals with mild to moderate AD.	It is recommended to conduct larger-scale and long-term clinical studies.	[370]
**Home Exercise Programs for Nursing Skills Training**	Alleviate patients' frailty and behavioral disorders.	Randomized clinical trials	153 patients	Three months	The combination of exercise training and behavioral management skills training for caregivers has improved the physical health and depressive symptoms of patients with AD.	Engaging in physical exercise within a home environment may pose risks of falls or injuries. Caregivers must ensure the safety of the exercise activities and, when necessary, provide assistive devices or personnel assistance.	[371]
**Souvenaid**	Souvenaid aims to enhance synaptogenesis and synaptic function.	Multinational randomized, controlled, double-blind, parallel-group trial.	259 patients	24 weeks	Souvenaid is well-tolerated and can improve memory performance with mild Alzheimer's disease, potentially supporting changes in synaptic activity by enhancing functional connectivity in the brain.	It is recommended to conduct larger-scale and long-term clinical studies.	[309]
**Family-based physical activity intervention**	Physical activity interventions can enhance physical and cognitive outcomes in older adults.	Randomized clinical trials	106 participants	24 months	Long-term adherence to physical activity is achievable and confers health benefits.	The conditions and needs of different patients vary significantly, necessitating a highly personalized approach to exercise interventions, thereby avoiding a one-size-fits-all methodology. Regular assessments and adjustments of the program are essential to accommodate the evolving circumstances of the patients.	[372]
**B vitamins**	Supplementation of B vitamins may slow cognitive decline in patients with MCI characterized by significant atrophy of the left frontal lobe.	Randomized clinical trials	279 individuals with Mild Cognitive Impairment (MCI)	24 months	Patients with a left frontal lobe atrophy rate higher than the median exhibited lower CDR_SOB scores (indicating better cognitive function) following supplementation with B vitamins, whereas no such effect was observed in patients with a lower atrophy rate.	It is recommended to conduct larger-scale and long-term clinical studies.	[307]

A variety of interventions are being explored as potential clinical treatments for AD, targeting different aspects of the disease pathology, such as neurodegeneration, inflammation, and cognitive decline. Leptin and Insulin-like Growth Factor-1 (IGF-1) have shown promise in promoting neuroprotection and supporting neuronal growth. MAPTRx, a therapy targeting tau pathology, and Aducanumab, an antibody that clears amyloid plaques, are focused on reducing key AD biomarkers. Brexpiprazole, an atypical antipsychotic, and Masitinib, a tyrosine kinase inhibitor, aim to manage neuroinflammation. Posiphen and CT1812 explore mechanisms to reduce amyloid-beta production, while Semorinemab and Donanemab target tau and amyloid accumulation, respectively. Other interventions, like Plasma exchange and Fortasyn Connect, are designed to modulate neuroinflammation and improve cognitive function. Intranasal Insulin Therapy and Intracerebroventricular injection of human umbilical cord blood-derived mesenchymal stem cells (hUCB-MSCs) aim to enhance insulin signalling and promote neurogenesis. Non-pharmacological treatments, such as Aerobic cycling, home exercise programs, and Family-based physical activity interventions, focus on improving physical health and cognitive performance. Supplements like Docosahexaenoic acid (DHA) and B vitamins are being investigated for their potential to support brain functionNeuroEPO plus, plus Tilavonemab, Gamma transcranial alternating current stimulation (tACS), also) represent novel approaches to modulate brain activity and improve neuroplasticity. Collectively, these interventions represent a broad spectrum of strategies aimed at alleviating symptoms, slowing disease progression, and improving the quality of life for AD patients.

Neprilysin is physiologically responsible for the clearance of Aβ in the body and the accumulation of this. Protein is considered a critical factor in the pathological processes of AD. Sacubitril/valsartan, a commonly used neprilysin inhibitor, is a combination drug primarily utilized in the treatment of heart failure. Sacubitril may increase plasma Aβ levels by inhibiting neprilysin activity, raising concerns regarding its potential implications for AD risk [274]. Posiphen is an investigational drug that reduces APP translation by binding to the iron-responsive element (IRE) within the APP mRNA. A double-masked, phase 1b, randomized dose-escalation clinical trial was conducted involving participants with mild cognitive impairment or early-stage AD, confirmed by low CSF Aβ42/40 ratios, randomly assigned to receive either Posiphen or a placebo. Pre-treatment assessments included lumbar puncture for CSF sample collection. After 21-23 days of treatment with Posiphen or placebo, participants underwent CSF catheter placement, intravenous administration of 13C6-leucine, and CSF sampling within 36 hours. Although the sample size was small, the study provided additional support for the safety and pharmacokinetic data of Posiphen [275].

The results of the randomized, placebo-controlled, double-blind trial of the tau-targeting drug Semorinemab indicate that it failed to slow functional decline in patients with moderate to mild AD, suggesting the need for further exploration of its effects at different stages of the disease or in combination with other therapeutic modalities [276]. Despite its notable target engagement, in a Phase II randomized double-masked placebo-controlled study, Tilavonemab did not demonstrate significant therapeutic benefits in early AD patients. This suggests that monotherapy targeting tau protein may be insufficient to address the complex pathology of AD [277]. In a Phase 1B randomized, placebo-controlled trial of the tau-targeting antisense oligonucleotide MAPTRx, MAPTRx exhibited a dose-dependent reduction in tau protein levels in patients with mild AD, with effects lasting for 24 weeks, indicating its potential therapeutic efficacy as a pathological biomarker [278].

Regarding antipsychotic medications, two randomized, double-blind, placebo-controlled trials indicate that Brexpiprazole has potential efficacy in alleviating agitation symptoms in AD patients, particularly demonstrating improvement in the Clinical Global Impression-Severity scale (CGI-S); however, some comparisons did not achieve statistical significance [279, 280]. A phase 2/3 multicenter, randomized, double-masked, placebo-controlled parallel-group study. After 10 weeks of treatment with Brexprazole, improvement in the symptoms of AD was observed. The efficacy of Brexprazole at a dosage of 1 mg/day was confirmed for the first time [281].

NeuroEPO plus is a recombinant human erythropoietin that lacks erythropoietic activity and possesses a shorter plasma half-life due to its low sialic acid content. This medication can prevent oxidative damage, neuroinflammation, apoptosis, and cognitive deficits in AD models. Double-blind, randomized Phase II-III clinical trials indicate that NeuroEPO plus improves cognitive assessments in patients with mild to moderate AD over 48 weeks, demonstrating good safety profiles; however, larger-scale trials are required to validate its efficacy [282]. CT1812 is a novel brain-penetrant sigma-2 receptor ligand that can interfere with binding Aβ oligomers to neurons. In the phase 1/2 study, CT1812 did not demonstrate significant differences in cognitive clinical scores; however, it showed a positive trend in MRI volumetric measures indicating tissue protection, thereby supporting its further clinical development [283].Additionally, Neuroad™ demonstrates efficacy and safety in the treatment of AD. It is a promising therapeutic agent [284]. A multicenter randomized placebo-controlled clinical trial indicates that methylphenidate is a safe and effective medication for addressing apathy in AD [285]. As a tyrosine kinase inhibitor, masitinib has shown potential benefits for patients with mild to moderate AD in a 12-week trial, and more extensive confirmatory studies are currently underway [286].

The 2B/3 phase study indicates that plasma exchange combined with albumin replacement therapy may slow cognitive and functional decline in AD patients, demonstrating good safety and feasibility [287]. About cellular therapy, phase I clinical trial results suggest that the administration of three repeated doses of human umbilical cord blood-derived mesenchymal stem cells (MSChUCB-MSCs) via the lateral ventricle is both feasible and safe, showing favorable outcomes in functional results and amyloid levels, with an expanded study currently underway in phase IIA [288]. A double-masked, randomized phase I trial supports the safety of Lomecel-B in the treatment of AD, and preliminary assessments indicate potential efficacy, with plans for a phase II trial to validate its effects and mechanisms in the future [289].

Regarding hippocampal neurogenesis, adult hippocampal neurogenesis (AHN) endows the entire hippocampal circuitry with unparalleled plasticity. The dentate gyrus (DG), a component of the hippocampus, belongs to the limbic system and plays a crucial role in learning, memory, and emotional regulation. It is one of the most significant structures within the hippocampus, primarily functioning in hippocampal neurogenesis. The persistent presence of neurogenesis during both physiological and pathological aging in humans provides evidence of impaired neurogenesis associated with potential memory deficits in AD, which may be amenable to novel therapeutic strategies [34]. A double-masked, randomized controlled single-center trial indicates that 40Hz transcranial alternating current stimulation (tACS) can enhance theta-gamma coupling and neuronal activity within the hippocampus, revealing a potential mechanistic model for therapeutic intervention [290].

Although some interventions have demonstrated potential effects in early studies, most remain in the clinical trial phase and face challenges such as unclear safety, efficacy, and mechanisms. Future research should focus on multimodal intervention strategies that integrate pharmacological and non-pharmacological approaches to address the complex pathological mechanisms of AD. Furthermore, it is essential to increase sample sizes, extend follow-up durations, and explore individualized treatment plans to achieve more effective prevention and treatment of AD.

### 8.2 Therapeutic markers

Therapeutic markers as shown in Table 2. Although biomarkers can detect amyloid and tau pathologies, relying solely on these markers for diagnosis presents numerous challenges, such as low accuracy and confusion with the pathologies of other neurodegenerative diseases [2]. The levels of Aβ42, the Aβ40/Aβ42 ratio, phosphorylated tau (p-tau), and the results of amyloid PET imaging (such as the uptake of radiotracers) in CSF can reflect the accumulation of amyloid plaques in the brain [291]; however, they do not necessarily predict whether an individual will exhibit clinical symptoms (such as mild cognitive impairment or dementia) in the future, particularly within cognitively normal populations. Asymptomatic individuals who test positive for biomarkers should be regarded as a "risk group" rather than being directly diagnosed with AD.

**Table 2:** Clinical markers in certain potential Alzheimer's disease patients.

Biomarkers	Sample source	Clinical significance	Ref.
**Abdominal aortic calcification (AAC) levels in older women**	Transverse Spine Image (LSI)	Women with moderate-to-high AAC have double the risk of dementia in later life, and this risk is not related to cardiovascular risk factors and apolipoprotein E (APOE) genotype.	[299]
**Adenosine receptor A1 (A1R)**	AD patient-derived neurons	This upregulation is closely associated with tau protein pathology.	[293]
**ApoJ/Clusterin**	CSF and same-day EDTA plasma	Compared to normal controls, CSF cholesterol efflux capacity (CEC) is significantly reduced in patients with MCI and shows poor correlation with plasma CEC. CSF ApoJ/Clusterin levels are also significantly lower in MCI patients and are strongly associated with CSF CEC. However, CSF ApoE is not related to CSF CEC.	[373]
**CXCL-11**	CSF	CXCL-11 peaks at an early stage and can be used to identify patients with MCI. As the disease progresses, CXCL-11 levels decrease slightly.	[38]
**Exosomes (EVs) membrane antigens and miRNAs encapsulated therein**	Blood	Compared to patients with mild AD, levels of myelin oligodendrocyte glycoprotein (MOG) in EVs are significantly higher in moderate and severe AD patients. Levels of EVs expressing the axonal glycoprotein CD171 are significantly elevated in severe AD patients compared to healthy controls. Endothelial-derived EVs are also increased in AD patients. Compared to EVs from healthy controls, EVs in AD patients show more than a twofold increase in inflammatory cytokines and a 50% reduction in growth factors. The levels of let-7g-5p, miR-126-3p, miR-142-3p, miR-146a-5p, and miR-223-3p are associated with disease severity.	[304]
**LCN2**	CSF	LCN2 plays an early role in the pathogenesis of Alzheimer's disease and may serve as an early blood biomarker for Aβ pathology. LCN2 can differentiate VaD from AD with high accuracy, and CSF LCN2 is a promising candidate biomarker for distinguishing VaD from neurodegenerative dementias.	[296]
**LCN2**	Pathological sections of autopsy brain samples	Elevated levels of Lcn2 in AD patients are associated with reactive gliosis and reduced neurogenesis.	[374]
**LCN2**	Plasma	Although LCN2 does not show significant differences in mixed dementia, its levels are indeed lower in rapidly progressing AD patients. LCN2 is not significantly associated with CSF biomarkers of neurodegeneration, AD-related pathology, cognitive status, APOE genotype, or the presence of white matter hyperintensities.	[375]
**LCN-2**	Plasma	Plasma LCN2 levels are higher in preclinical AD and are associated with CSF Aβ42 levels, while CSF tau and phosphorylated tau181 (p-tau181) levels are also elevated.	[295]
**LCN-2**	Plasma	Elevated levels of LCN2 in the blood can serve as an early predictor before MCI progresses to AD, potentially facilitating early intervention and treatment.	[297]
**miR-124-3p and miR-125b-5p**	Serum	Its increased expression may serve as a potential diagnostic biomarker for AD.	[292]
**NGAL (neutrophil gelatinase-associated lipoocalin)**	CSF	It reaches its peak at an early stage and can be used to identify patients with MCI.	[38]
**NGAL (neutrophil gelatinase-associated lipoocalin)**	Serum and CSF	A positive correlation exists between CSF NGAL and Aβ1-42. CSF NGAL has the potential to serve as a biomarker for predicting the conversion of MCI to AD dementia.	[39]
**Osteopontin (OPN)**	CSF	CSF osteopontin (OPN) levels can indicate early synaptic dysfunction.	[376]
**Osteopontin (OPN)**	Serum	Increased OPN levels are associated with neuroimaging markers of vascular cognitive impairment and neurodegeneration, and there is a correlation between OPN and inflammatory cytokines such as IL-6, IL-8, and TNF.	[303]
**Osteopontin (OPN)**	Serum and CSF	i) OPN levels in the CSF of AD patients are significantly elevated. ii) OPN levels are correlated with Mini-Mental State Examination (MMSE) scores; and iii) OPN levels are higher in the early stages of the disease (≤2 years). These findings support the role of OPN in the pathological processes of AD.	[302]
**sTREM1**	CSF	Although sTREM1 is more commonly associated with acute and infectious inflammation, its expression in AD patients may be related to the persistent inflammatory response of the disease.	[38]
**sTREM2**	CSF	It is considered an anti-inflammatory factor, with its concentration significantly increasing in late-stage AD patients, and it is associated with tau protein accumulation and the pathological processes of AD.	[38]
**tTau**	Serum	Elevated levels of tTau in AD patients are often associated with neuronal injury or death.	[292]
**Vascular endothelial growth factor (VEGF) and transforming growth factor-β1 (TGF-β1)**	Serum	The serum VEGF concentration in AD patients is significantly lower than in patients with (aMCI) and healthy controls. The VEGF level in aMCI patients is also significantly lower than in healthy controls. Additionally, TGF-β1 levels are significantly reduced in both AD and aMCI patients compared to controls. Serum VEGF/TGF-β1 levels are negatively correlated with Clinical Dementia Rating (CDR), while serum VEGF levels are positively correlated with TGF-β1 levels.	[377]

Clinical biomarkers for AD are crucial for early diagnosis, monitoring disease progression, and guiding treatment. Several biomarkers reflect different aspects of AD pathology, including inflammation, neurodegeneration, and vascular dysfunction. Notable biomarkers include Abdominal Aortic Calcification (AAC), which correlates with vascular damage and cognitive decline in older women, and Adenosine Receptor A1 (A1R), which modulates neuroinflammation. Apolipoprotein J (ApoJ) helps in amyloid-beta clearance, while CXCL-11 and Lipocalin-2 (LCN2) are linked to immune responses and neuroinflammation. Exosomes, containing membrane antigens and miRNAs, reflect cellular changes and may serve as non-invasive biomarkers. miR-124-3p and miR-125b-5p are involved in inflammation and neuronal function, while NGAL indicates neuroinflammatory stress. Osteopontin (OPN), sTREM1, and sTREM2 are markers of immune activation, and total tau (tTau) is a key marker of neuronal damage. Lastly, VEGF and TGF-β1 are associated with vascular dysfunction and brain remodeling. These biomarkers offer valuable insights into AD’s underlying mechanisms and therapeutic targets.

The Korean Alzheimer’s Disease Research Platform (K-ARPI) is advancing new strategies based on immune-inflammatory biomarkers to identify AD subpopulations and modulate toxic immune-inflammatory responses. The platform aims to develop novel drugs that can slow the progression of AD [271].

In patients with AD, the increased levels of miR-483-5p are significantly negatively correlated with the reduction in bone mineral density (T-Score), indicating its potential as a biomarker for assessing AD-related osteoporosis [100]. Additionally, serum miR-124-3p may serve as a promising diagnostic biomarker and a new therapeutic target for AD. The changes in expression levels of miR-124-3p and miR-125b-5p in the serum of Alzheimer's patients, particularly the increase in miR-124-3p, may be potential diagnostic biomarkers for AD [292]. Furthermore, it has been discovered that the expression of adenosine receptor A1 (A1R) is significantly upregulated in induced pluripotent stem (iPS) cells from AD patients, and this upregulation plays a critical role in tau protein pathology. This suggests that A1R may be a biomarker for monitoring AD progression and tracking changes in the disease state. It was also found that miR-133a-3p is downregulated in AD, while its target A1R is upregulated. These findings indicate that targeting miR-133a-3p may help restore normal adenosine A1 receptor signaling, potentially improving cognitive function in AD patients [293]. The pro-inflammatory proteins NGAL and CXCL-11 exhibit a significant increase in concentration during the early stages of the disease, effectively identifying patients with MCI. Conversely, the anti-inflammatory molecule sTREM2 reaches its highest concentration in the later stages of the disease, distinguishing AD patients from non-demented control groups.

Furthermore, sTREM2 significantly correlates with Tau and its phosphorylated form (pTau) within the AD group. These findings suggest that proteins secreted by dysfunctional microglia may play dual roles, detrimental and protective, in the pathological progression of AD [38]. The neurofilament light chain (NfL) changes may be closely associated with neuronal injury and death, independent of the most common risk factors such as age and sex. They can reflect the progression of various neurodegenerative diseases, thus being regarded as an effective biomarker for disease assessment. Elevations in NfL levels can typically be detected before the onset of clinical symptoms. Changes in NfL levels are consistent with pathological alterations observed in neuroimaging, such as brain atrophy and amyloid plaque deposition as revealed by MRI and PET scans. The strong correlation with these imaging biomarkers renders NfL a crucial adjunctive tool [31]. The Aβ42/40 ratio shows a negative correlation with the T2 values of the choroid plexus, meaning that a lower Aβ42/40 ratio is associated with higher T2 values in this region. This suggests that an increase in Aβ deposition may be linked to damage in the microstructure of the choroid plexus. On the other hand, pTau181 is positively correlated with both the T1 and T2 values of the choroid plexus, indicating that higher levels of pTau181 are associated with increased T1 and T2 values. This suggests that elevated levels of pTau181 may also be related to damage in the microstructure of the choroid plexus [294].

Research has identified elevated levels of LCN2 in CSF as a marker that helps distinguish AD from other neurodegenerative diseases, correlating with CSF amyloid-β42 levels [295-297]. Positron emission tomography (PET) imaging has been employed to measure in vivo upregulation of COX-2 using three clinically translatable tracers: [11C]TMI, [11C]MC1, and [18F]MTP. These radioligands exhibit excellent BBB permeability, providing potential tools for AD staging and therapy evaluation [298]. Non-invasive diagnostic strategies, such as combining bone density testing with measuring abdominal aortic calcification (AAC) levels, have emerged as methods to identify individuals at high risk for late-life dementia [299]. OCN has been found to exert protective effects against AD [300], potentially by regulating blood glucose and cholesterol levels [301]. Osteopontin (OPN) levels in the CSF are significantly elevated in AD patients, particularly during the early stages, indicating its involvement in AD pathology [302]. In a cross-sectional study based on a cohort from a memory clinic in Singapore, 80 subjects without cognitive impairment, 160 subjects with Cognitive Impairment Not Dementia (CIND), and 144 subjects with dementia were included. The increase in OPN was significantly associated with neuroimaging markers of Cerebrovascular Disease (CEVD) and neurodegeneration, including cortical infarcts, fissures, white matter hyperintensities, and brain atrophy. OPN was also correlated with elevated IL-6, IL-8, and TNF levels. Our findings suggest that OPN may play a role in Vascular Cognitive Impairment (VCI) and neurodegenerative dementia [303].

Exosomal analysis in AD patients reveals significantly increased levels of myelin oligodendrocyte glycoprotein (MOG) and axonal glycoprotein (CD171), as well as elevated levels of inflammatory cytokines [304]. Additionally, live neuroimaging techniques utilizing X-ray CT and gold nanoparticle tracers have successfully tracked exosome migration and targeting patterns under various neuropathological conditions, particularly in inflammation-driven pathological regions [305].

Age-related urinary proteomic biomarkers (UPP), particularly those referred to UPPost-age, are associated with the risk of osteoporosis. These peptides are age-dependent and primarily derived from collagen (COL1A1, COL1A2, COL2A1, etc.), IGF2 (Insulin-like Growth Factor II), and MGP (Matrix Gla Protein). An analysis of data from 706 participants revealed that an increase in UPPost-age significantly correlates with an elevated risk of osteoporosis. Specifically, for all participants, every 10-year increase in UPPost-age is associated with a 36% increase in the HR of osteoporosis, while in females, this increase reaches 60%. Furthermore, UPPost-age also demonstrates a significant positive correlation with the risk of fractures. As a urinary biomarker, UPPost-age indicates that this metric can be utilized in clinical practice to assess the risk of osteoporosis. Given that osteoporosis is often challenging to identify early in clinical settings, the application of UPPost-age offers new possibilities for early screening and intervention, thereby aiding in the prevention of osteoporosis-related fractures. Additionally, this biomarker has been registered in Germany and the European Union as an in vitro diagnostic tool (IVD), signifying its potential for widespread application in clinical environments, providing crucial information regarding osteoporosis risk to patients and healthcare providers [306].

These biomarkers correlate with disease severity, suggesting their relevance in tracking AD progression and identifying therapeutic targets. Together, these findings emphasize the potential of biomarkers and advanced imaging technologies in the early de tection, staging, and treatment of Alzheimer’s disease.

### 8.3 Dietary therapy

Dietary factors such as flavonoids, probiotics, and various minerals have shown potential in reducing neuroinflammation and improving cognitive function in preclinical models [23]. However, these studies remain fragmented and lack systematic validation. Although some dietary intervention clinical trials have suggested that composite nutritional patterns may help alleviate inflammation and lower the risk of AD, most of these trials are observational studies or small-scale experiments. More extensive randomized controlled trials are needed to confirm these findings [23]. Key inflammatory pathways associated with AD development include gut microbiota interactions, fatty acid metabolism, and dietary advanced glycation end products (d-AGEs). Potential dietary factors targeting inflammation include omega-3 fatty acids, flavonoids, polyphenols, vitamin D3, and probiotics. These compounds are believed to improve inflammatory states by modulating immune responses and reducing pro-inflammatory cytokine levels [23].

Supplementation with B vitamins may slow cognitive decline in patients with MCI who exhibit significant atrophy in the left frontal lobe. This finding suggests that patients with atrophy in specific brain regions may benefit more from B vitamin supplementation. Although B vitamin supplementation does not significantly impact cognitive function, studies indicate it can reduce homocysteine levels. Elevated homocysteine levels are associated with an increased risk of cognitive decline; therefore, B vitamins may indirectly enhance brain health by lowering homocysteine levels [307]. Randomized placebo-controlled trials indicate that the use of omega-3 supplements at daily doses equal to or less than 1 gram has limited effects on brain health in AD, particularly among APOE4 carriers, thereby underscoring the necessity for research involving high-dose DHA [308]. Supplementation with B vitamins may slow cognitive decline in patients with mild cognitive impairment who exhibit significant atrophy in the left frontal lobe, while the effects are not significant in patients with less atrophy, suggesting that genotype and brain structure play a crucial role in the efficacy of the intervention [307]. Souvenaid is a medical food specifically designed for patients with Alzheimer's disease, aimed at slowing cognitive decline by providing essential nutrients that support the formation and maintenance of brain synapses. In a randomized, controlled, double-blind, parallel-group multinational trial, Souvenaid was well tolerated and demonstrated improvements in memory performance among patients with mild Alzheimer's disease, potentially supporting changes in synaptic activity through enhanced brain functional connectivity [309].

Fortasyn Connect is a medical food formulation designed to support cognitive function in patients with AD. Its primary components include ω-3 fatty acids, ω-6 fatty acids, choline, uridine monophosphate, vitamins, and minerals. In a randomized, double-blind, placebo-controlled trial lasting 36 months, Fortasyn Connect significantly improved cognitive function in patients with prodromal AD, slowed brain atrophy, and demonstrated significant improvement on the Clinical Dementia Rating Scale [310].

Additionally, intermittent fasting and high-protein diets have been found to alleviate memory impairments and bone joint symptoms, potentially through mechanisms such as reducing hippocampal Aβ deposition, lowering pro-inflammatory cytokine expression, and increasing lean body mass [311]. Green tea and its primary active component, epigallocatechin-3-gallate (EGCG), have emerged as promising dietary interventions for AD. EGCG exerts antioxidant effects, regulates ferroptosis and microglia-induced cytotoxicity, inhibits tau hyperphosphorylation and aggregation, and promotes non-amyloidogenic processing of APP, thereby preventing the formation and accumulation of Aβ [312]. Specific minerals have also been implicated in AD risk modulation. Copper intake has been associated with a reduced risk of AD, while magnesium supplementation may increase the risk of rheumatoid arthritis and bipolar disorder [313]. Type I interferons have been identified as potential anti-inflammatory lead molecules, capable of activating gut-associated lymphoid tissue and influencing brain inflammation, though further clinical validation is required [314].

In conclusion, while dietary interventions hold promise in modulating neuroinflammation and mitigating AD risk, more comprehensive and large-scale clinical studies are needed to establish their efficacy and underlying mechanisms.

### 8.4 Natural Anti-inflammatory Products

Natural Anti-inflammatory Products, as shown in Table 3. Oxidative stress and inflammatory responses are critical drivers in neurodegenerative diseases and neuro-inflammation pathophysiology. Numerous natural and synthetic compounds have demonstrated significant neuroprotective and anti-inflammatory effects by targeting specific enzymes and signaling pathways [315, 316]. Some studies are conducted solely at the animal level, while clinical trials require time to develop specific research.

**Table 4:** Commonly used therapeutic target drugs in animal and cell experiments.

Classification	Drugs	Therapeutic targets	Experimental models	Ref.
**COX-2 inhibitors**	Celecoxib	Celecoxib improved the resting-state functional connectivity (RSFC) in the default mode network (DMN). The inhibition of p65 phosphorylation negated the enhancement of connexin 43 (CX43).	LPS was administered to the prefrontal cortex (PFC) to establish a model of major depressive disorder (MDD).	[344]
**COX-2 inhibitors**	Celecoxib	Celecoxib inhibition of COX-2 improves neurobehavioral responses.	15% kaolin-induced seven-day-old male Wistar rats	[345]
**CSF1R inhibitors**	PLX3397	The number of microglial cells in the brain is significantly reduced, including those associated with cortical plaques, while the burden of Aβ plaques in the cortex is also markedly decreased.	5xFAD mouse, microglias	[362]
**CSF1R inhibitors**	PLX3397	The administration of PLX3397 can inhibit amyloid pathology while simultaneously preserving dopamine signaling.	5xFAD mouse	[364]
**CSF1R inhibitors**	PLX3397	A significant reduction in soluble protofibrillar amyloid oligomers in brain lysates, a depletion of soluble fibrinogen-derived oligomers in plasma, and an improvement in cognitive function measured through fear modulation tests.	5xFAD mouse	[361]
**CSF1R inhibitors**	PLX5622	Low levels of CSF1R inhibition can prevent the binding of microglia to plaques and improve cognitive function.	3xTg-AD mouse	[366]
**NLRP3 inhibitors**	MCC950	The overexpression of NLRP3, caspase-1, and GSDMD associated with pyroptosis in SAMP8 mouse neurons was suppressed, indicating that the neuronal pyroptosis induced by the NLRP3/caspase-1/GSDMD axis is a significant factor contributing to neuronal loss in AD.	Accelerated Aging Mouse (SAMP8)	[228]
**NMDA receptor antagonists**	Memantine	The reversal of the glutamate/NMDA receptor axis in the neuroinflammation process of JEV pathogenesis.	Mouse infected with JEV	[378]
**NMDA receptor antagonists**	Memantine	Improved the profile of inflammatory cytokines, reducing the levels of IL-6 and TNF-α.	Male Sprague Dawley rats PTSD models	[379]
**NMDA receptor antagonists**	NMDARS	Blocking the NMDAR/Ca2+/Calpain-mediated dysregulation of BDNF/TrkB signaling in the pathogenesis of POCD.	C57BL POCD animal models	[380]
**NMDA receptor antagonists**	Memantine	Inhibition of microglial activation by blocking microglial Kir2.1 channels.	Mouse models of dynamic pain hypersensitivity	[381]
**NMDA receptor antagonists**	Memantine	The activation of the transcription factor NF-κB was reversed by preventing the phosphorylation and degradation of its inhibitor IκBα. This represents a novel anti-inflammatory mechanism driven by the neuroprotective effects of memantine mediated by endothelial cells.	Monocyte THP-1 cells and human brain microvascular endothelial cells (HBMVE)	[382]
**NMDA receptor antagonists**	Memantine	Improved the reduction of neurotrophic factors induced by STZ (BDNF, GDNF) and insulin resistance dysfunction, and decreased the levels of inflammatory markers, nuclear factor kappa-B translocation, glial fibrillary acidic protein, cyclooxygenase-2, tumor necrosis factor-α, and oxidative-nitrosative stress.	STZ intervention in astrocytes.	[383]
**NMDA receptor antagonists**	Memantine	Blocking the Kir2.1 channel	RAW 264.7 macrophages and BV2 microglial cells	[384]
**NMDA receptor antagonists**	Memantine	By preventing the activation of microglia, there is an increase in the release of neurotrophic factors from astrocytes and anti-inflammatory agents.	LPS-induced microglial models, dopaminergic (DA) neurons	[385]
**NMDA receptor antagonists & NSAIDs**	Memantine and Ibuprofen	Only USD can reverse the changes in NMDA receptor subunit expression induced by STZ.	(ICV STZ) injection	[386]
**TNF-α** **inhibitors**	Etanercept (ETN)	It reduced the levels of inflammatory cytokines and inhibited the activation of the C-JUN N-terminal kinase (JNK) and nuclear factor-kappa B (NF-κB) pathways.	APPSWE/PS1M146V/TAUP301L transgenic (AD) mouse	[350]
**TNF- α inhibitors**	Adalimumab	It alleviated neuronal loss in the hippocampus of VAD rats. Furthermore, adalimumab significantly reduced microglial activation and reversed the M1/M2 polarization in VAD rats. It also inhibited the activity of NF-κB.	VAD rats model	[348]
**TNF-α inhibitors**	TFRMAB-TNFR (BBB - permeable) and non-BBB permeable (Etanercept)	Improved neuronal health, increased PSD95 expression, and alleviated the loss of hippocampal neurons.	Male and female PS19 mouse	[352]
**TNF- α inhibitors**	Intraperitoneal injection of infliximab	It alleviated joint inflammation and reduced paw edema. Infliximab improved the trabecular microstructure and reversed the tendinopathy induced by collagen-induced arthritis (CIA). The levels of TNF-α and IL-23 significantly decreased.	The collagen-induced arthritis (CIA) model induces inflammation in rats.	[149]
**Targeted glioma inhibitors**	Inhibition of astrocytic α2-NKA	α2-NKA influences neuroinflammation by regulating the secretion of pro-inflammatory cytokines and IL-6.	Tau-tg mouse	[78]
**Sesquiterpene lactone derivatives**	ACT001	Inhibition of AKT phosphorylation restricts the nuclear translocation of NFκB in microglia, leading to a reduction in NLRP3 inflammasome activation, ultimately downregulating the neuroinflammatory response of microglia. This is achieved by decreasing trauma-induced microglial activation, thereby alleviating the extent of blood-brain barrier integrity impairment and mitigating the severity of motor function deficits following TBI.	TBI mouse model, LPS-induced BV2 cells, bEnd.3 cells	[324]
	ACT001	Inhibition of AKT phosphorylation suppresses the nuclear translocation of NFκB and reduces the activation of the NLRP3 inflammasome.	BV-2 microglial cells Sprague-Dawley rats	[324]
**Dipeptide**	Carnosine	Protecting BV-2 microglial cells from the oxidative stress and inflammation induced by Aβ1-42 oligomers involves reducing endogenous nitric oxide and reactive oxygen species levels, decreasing the expression of iNOS and Nox enzymes, and diminishing the secretion of pro-inflammatory cytokines such as IL-1β. Furthermore, carnosine can enhance the expression and secretion of TGF-β1 and improve IL-10 levels, thereby preventing amyloid β-induced neurodegenerative diseases.	BV-2 microglial cells	[387]
**Synthesis of Sesquiterpene Lactones**	ACT001	Inhibition of AKT phosphorylation suppresses the nuclear translocation of NFκB and reduces the activation of the NLRP3 inflammasome.	BV-2 microglial cells Sprague-Dawley rats	[324]
**Synthetic multi-target inhibitors**	Enol-ketone compounds	Inhibitors such as carbonic anhydrase, monoamine oxidase, and cholinesterase.	Rat astrocytes	[319]
**Synthetic Coumarin Derivatives**	Alkyl Substituted Coumarins	Mitigation of oxidative stress-induced neuroinflammation and IL-6 secretion.	Astrocytes stimulated by LPS in rats	[319]
**Gene therapy**	miR-133a-3p	Therapeutic interventions targeting miR-133a-3p may facilitate the restoration of normal adenosine A1 receptor signaling, thereby contributing to the improvement of cognitive function in AD.	3×TG mouse	[293]
**Gene therapy**	miR-135-5p	By inhibiting astrocyte-mediated neuroinflammation and blocking the JAK2/STAT3 signaling pathway, miR-135-5p can potentially reduce BCP in mice.	The potential role in the BCP mouse model, which is established through tumor cell implantation (TCI) in the femoral head.	[388]
**Hormones**	Osteocalcin	Reduce Aβ burden and upregulate glial glycolysis.	APP/PS1 transgenic Alzheimer's disease mouse model	[389]
**Anti-inflammatory agent**	UB-ALT-EV	It is capable of reducing the gene expression of microglial activation markers Trem2 and NF-κB, while the inflammatory cytokines IL-1β, IFN-γ, CCL2, and CCL3 are downregulated.	5XFAD mouse	[390]
**Anti-inflammatory agent**	amlexanox	It exerts anti-inflammatory effects by downregulating the NF-κB and STAT3 signaling pathways, and the combination with the selective STAT3 inhibitor SPI demonstrates high efficacy in inhibiting neurotoxicity and pro-inflammatory mediators.	LPS-induced bone marrow-derived macrophages, mouse BV2 cells, and human HMC3 microglial cells.	[391]
**Antioxidants**	Antioxidant N-acetyl-L-cysteine (NAC)	Antioxidant, anti-inflammatory, and cytoprotective effects.	Tg2576 mouse	[214]
**Steroid-enriched extract**	AB(ABS)Steroid-Enriched Fraction of Achyranthes bidentata	Regulating the ERK pathway, NF-κB phosphorylation reverses neurooxidative damage and neuroinflammation.	Aβ1-40 induced AD rat models	[392]
**Ion channel blockers**	Inhibition of the calcium-activated potassium channel KCa3.1 in astrocytes.	Blocking KCa3.1 can significantly reduce neuroinflammation and glial cell activation, thereby decreasing neuronal loss and memory deficits.	TgAPP/PS1 mouse	[393]
**Nanomaterials**	TPP-MoS2 QDs	The ability to transition microglia from the pro-inflammatory M1 phenotype to the anti-inflammatory M2 phenotype can reduce Aβ aggregation-mediated neurotoxicity and eliminate Aβ aggregates in Alzheimer's disease mouse models.	BV-2 TgAPP/PS1 mouse	[394]
**Metal**	Lithium	Chronic lithium treatment attenuated autophagy activation in this Alzheimer's disease mouse model, reducing the production of amyloid-β and the formation of senile plaques. It modulated GSK3β activity.	Aged double transgenic mouse (AβPPSWE/PS1A246E)	[395]
**Tryptophan derivatives**	Two novel tryptophan (TRYP) derivatives.	Restoration of neuronal cells in the CA3 and CA1 regions of the hippocampus effectively ameliorated learning and memory impairments in a scopolamine-induced AD mouse model.	Scopolamine-induced ADmouse model	[396]
**Dual enzyme inhibitors**	TPPU is treated in combination with a low dose of 6-chloratacrine	Increased the ratio of phosphorylated cAMP response element-binding protein (p-CREB), brain-derived neurotrophic factor (BDNF), and postsynaptic density protein 95 (PSD95), while simultaneously reducing the expression of neuroinflammatory genes.	Accelerated aging mouse model (SAMP8), 5xFAD mouse model.	[397]
**Dual enzyme inhibitors**	6-Chloro-phenylalanine (Huprine)-TPPU hybrid as a dual inhibitor of enzymes.	After low-dose chronic oral administration, it can improve memory, synaptic plasticity, and neuroinflammation in a mouse model of Alzheimer's disease.	AD mouse model	[398]
**Dual enzyme inhibitors**	The structure of the cholinesterase inhibitor huprine Y in conjunction with the antioxidant capsaicin.	The progression of Alzheimer's disease-like pathology was delayed, with a significant reduction in the hippocampal Aβ42/Aβ40 ratio, an increase in baseline synaptic efficacy, and a notable decrease in oxidative stress and neuroinflammation in the hippocampal region.	10-month-old APP/PS1 mouse	[399]
**Dual enzyme inhibitors**	Acetylcholinesterase inhibitors donepezil and rivastigmine.	Donepezil inhibited the phosphorylation of the AKT/MAPK signaling pathway, NLRP3 inflammasome, and transcription factors NF-kB/STAT3 induced by LPS, thereby reducing neuroinflammatory responses. Donepezil significantly alleviated LPS-induced microglial activation, microglial density/morphology, and levels of pro-inflammatory cytokines COX-2 and IL-6. In the Alzheimer's disease mouse model (5xFAD mice), Donepezil markedly decreased the activation, density, and morphology of Aβ-induced microglia and astrocytes.	LPS-induced BV2 microglial cells, 5xFAD mice	[400]
**Dual enzyme inhibitors**	Based on the active site of peptide-based ACHE inhibitors, an octapeptide.	Promotes neurite outgrowth, stabilizes intracellular microtubules, and confers significant neuroprotective effects following the withdrawal of nerve growth factor (NGF), thereby demonstrating additional potential as a microtubule stabilizer.	PC12-derived neurons	[334]
**Dual enzyme inhibitors**	2,3-Dihydro-1H-Inden-1-ones as dual PDE4/AChE inhibitors, 12C.	Exhibit more effective anti-neuroinflammatory properties.	AD model mouse	[398]
**Natural products**	Pinitol	Activation of iNOS, COX-2, and NF-κB, etc.	BV2 microglia cells	[321]
**Natural polyphenols**	Curcumin	By reducing inflammatory signaling through IL-1β and COX-2, levels of Aβ plaques and tau tangles are diminished.	Wistar Rats	[401]
**Natural compounds**	Osthole (OST) and notopterol (NOT)	More effectively inhibited the release of nitric oxide (NO) and reactive oxygen species (ROS).	Zebrafish AD/OP comorbidity models	[402]
**Natural flavonoids**	Natural Isoquercitrin (IQ)	Reduced the production of NO and PGE2, as well as the release of TNF-α and IL-1β, and the levels of oxidative stress, while inhibiting the activation of the NF-κB	BV2 microglia cells	[322]
**Natural flavonoids**	Baicalin (BA)	Regulation of the TLR4/myeloid differentiation primary response 88 (MyD88)/NF-κB pathway.	BV2 microglia cells	[323]
**Natural flavonoids**	Myricetin(Myr)	Specifically inhibit the activation of the p38 MAPK pathway while restricting the excessive activation of microglia.	3×TG-AD mouse	[326]
**Natural carotenoids**	Lycopene	Reduced the activity of pro-inflammatory cytokines TNF-α, TGF-β, IL-1β, as well as NF-κB and caspase-3 in the brain.	Wistar Rats	[328]
**Natural Alkaloids**	Berberine (BBR)	Enhancing the viability of bone marrow-derived mesenchymal stem cells (BMSCs) and the secretion of nerve growth factor (NGF) and brain-derived neurotrophic factor (BDNF).	Bone marrow mesenchymal stem cells (BMSCs)	[403]
**Natural extracts**	GJ-4	Regulation of the phosphatidylinositol 3-kinase/AKT (PI3K/AKT) signaling pathway activation inhibits neuroinflammatory responses in the brain.	APP/PS1 transgenic mice	[329]
**Natural extracts**	Zanthoxylum zanthoxyloides	Inhibition of NF-κB and activation of the NLRP3 inflammasome.	BV2 microglia cells	[404]
**Natural extracts**	Humulus lupulus L. (Hops)	The expression of antioxidant-related proteins NRF2, HO-1, NQO1, FOXO1, and SOD-2 was increased to prevent advanced osteoporosis by inhibiting Aβ deposition and oxidative stress.	APP/PS1 Osteoblasts	[405]
**Natural saponins**	Saikosaponins B2(SSB2)	Exhibits anti-tumor and anti-neuroinflammatory effects in a GPX4-dependent manner through the TLR4/NF-κB pathway.	ICR mouse	[327]
**Natural fatty acids**	10-Hydroxydecanoic Acid (10-HDAA)	Weakened the activation of the NF-κB pathway, subsequently targeting PTGS-1/2.	BV2 microglia cells	[406]
**Signal pathway inhibitors**	Wnt signaling inhibitor Wnt-C59	The anti-inflammatory effect of Wnt-C59 is achieved by reducing the intracellular levels of β-catenin and its interaction with NF-κB, thereby inhibiting NF-κB activity and the expression of pro-inflammatory cytokines.	LPS stimulated epithelial cells and macrophages.	[407]
**Acetylcholinesterase inhibitors**	Huperzine A, HupA	The inhibition of AchE resulted in a reduction of various pro-inflammatory cytokines, including iNOS, IL-1β, IL-18, CD16, and TNF-α.	CPZ mouse	[408, 320]
**Acetylcholinesterase inhibitors**	Donepezil	In OVX rats, no significant bone effects of donepezil were observed.	Ovariectomized (OVX) rats	[409]
**Acetylcholinesterase inhibitors**	AChEIs	Inhibiting RANKL-induced activation of MAPK and NFATc1 during the process of osteoclastogenesis, thereby increasing bone mineral density.	Ovariectomy-induced osteoporosis rat model	[410]
**Butyrylcholinesterase inhibitors**	Butyrylcholinesterase (Buche) compound 7p	Mitigates cognitive and memory impairment in scopolamine-induced mouse models, showing comparable effects to Rivastigmine	Scopolamine-induced mouse models	[411]
**Acetylcholinesterase inhibitors**	Huperzine A, HupA	The mRNA levels of various anti-inflammatory cytokines (ARG1, CD206) were increased, while the levels of different pro-inflammatory cytokines (iNOS, IL-1β, IL-6, IL-18, CD16, and TNF-α) were decreased.	Cuprizone (CPZ)-induced multiple sclerosis mouse model	[320]
**Acetylcholinesterase inhibitors**	The benzylpiperidine moiety was fused into the 1,2,4-oxadiazole core of the ACHE inhibitor donepezil, 15A.	Upregulation of NRF2 and its downstream proteins HO-1, NQO1, and GCLM at both the protein and transcriptional levels was observed, along with the promotion of NRF2 translocation from the cytoplasm to the nucleus. This exhibited significant neuroprotective functions by safeguarding cells against damage induced by H2O2 and Aβ1-42 aggregation, while also exerting antioxidant stress and anti-inflammatory activities through the reduction of ROS and pro-inflammatory cytokine production.	mouse	[412]
**Traditional Chinese Medicine Formulations**	Liuweidihuang (LW)	Inhibited glial activation and neuroinflammation (COX-2, IL-1β, IL-6, and TNF-α).	SAMP8 mouse	[325]
**Traditional Chinese Medicine Formulations**	BJTW	BJTW can alleviate D-galactose-induced osteoporosis by modulating the levels of alkaline phosphatase, osteocalcin, osteoprotegerin, and receptor activator of nuclear factor kappa B. Additionally, BJTW enhances the levels of catalase and glutathione peroxidase in serum, reduces the content of malondialdehyde in the hippocampal region, and increases the expression of Forkhead O1.	D-galactose-induced mouse model	[413]

Potential therapeutic targets include non-steroidal anti-inflammatory drugs, TNF-α inhibitors, interleukin modulators, and CSF1R inhibitors. The table content refers to experiments conducted on both animal and cellular levels.

Natural anti-inflammatory compounds such as curcumin, resveratrol, quercetin, and rosmarinic acid have shown promising effects in preclinical studies [317, 318]. Curcumin, for instance, has advanced to early clinical trials, demonstrating safety and efficacy [317]. Enolones, by inhibiting enzymes such as carbonic anhydrase, monoamine oxidase (MAO), and AChE, reduce oxidative stress and inflammation, creating a favorable environment for neuronal survival [319]. Huperzine A (HupA), derived from *Huperzia serrata*, inhibits AChE, increasing acetylcholine levels in the synaptic cleft, thereby improving neurotransmission and alleviating cognitive deficits in AD [320]. It also modulates anti-inflammatory cytokines like Arg1 and CD206 while reducing pro-inflammatory cytokines such as iNOS, IL-1β, and TNF-α [320].

Other compounds, including pinitol (a methylated derivative of D-chiro-inositol) [321], acylated IQ derivatives (compounds 9a-9c) [322], baicalin (BA) [323], ACT001 [324], and Liuwei Dihuang (LW) [325], effectively attenuate neuroinflammation by suppressing pro-inflammatory cytokines (e.g., TNF-α, IL-1β, IL-6) and enzymes like iNOS and COX-2. Most of these compounds act via inhibition of the NF-κB signaling pathway. For instance, pinitol blocks IκBα phosphorylation and degradation [321], baicalin regulates the TLR4/MyD88/NF-κB axis [323], and ACT001 inhibits NF-κB nuclear translocation by suppressing AKT phosphorylation [324].

Myricetin specifically inhibits the p38 MAPK pathway to prevent microglial overactivation and reduce neuroinflammation [326]. Saikosaponin B2 protects neurons from endoplasmic reticulum stress by inhibiting ferroptosis and maintaining calcium homeostasis through the TLR4/NF-κB and GPX4-dependent pathways [327]. Lycopene offers neuroprotection by mitigating oxidative stress and enhancing mitochondrial function [328]. GJ-4 regulates the PI3K/AKT signaling pathway, suppressing neuroinflammatory responses and subsequent inflammatory cytokine release [329].

These compounds represent a multifaceted approach to targeting oxidative stress and inflammation, providing a promising foundation for the development of novel therapeutic strategies for neurodegenerative diseases.

### 8.5 Exosomes Deliver Drugs

As shown in Table 3. Exosome-based drug delivery is emerging as a promising strategy for treating AD [330, 331]. Exosomes can cross the BBB efficiently, delivering therapeutic agents or molecules directly to the brain [332-334]. Recent studies have highlighted the neuroprotective properties of exosomes derived from bone marrow mesenchymal stem cells (BM-MSCs) and adipose tissue-derived mesenchymal stem cells (ADSCs). These exosomes have been shown to ameliorate AD pathology and enhance cognitive function [332, 335].

One key mechanism involves miRNAs carried within exosomes, such as miR-146a, which regulate astrocytic functions, promote synaptogenesis, and improve cognitive deficits [336]. Additionally, exosome-mediated delivery of the SphK/S1P signaling pathway has been found to reduce Aβ deposition and enhance cognitive performance in AD mouse models [47]. Nasal administration of MSC-derived exosomes has been reported to decrease co-localization of GFAP and Aβ plaques in the brains of 5XFAD mice, thereby mitigating pathological changes associated with AD [337].

Furthermore, ADSC-derived exosomes carrying enzymatically active neprilysin (NEP) have demonstrated the potential to reduce Aβ levels, underscoring their value in AD therapy. These findings suggest that as versatile drug delivery vehicles, exosomes hold significant potential for advancing AD treatment by addressing key pathological mechanisms and improving cognitive outcomes [338].

### 8.6 Nonsteroidal Anti-inflammatory Drugs (NSAIDs)

As shown in Table 3. Although observational studies suggest that non-steroidal anti-inflammatory drugs may reduce the risk of dementia, subsequent interventional study results have been disappointing [339]. Aspirin is considered beneficial for cardiovascular health in certain contexts; however, a randomized controlled trial (RCT) indicated that low-dose aspirin did not decrease the fracture risk in a normal elderly population and was instead associated with an increased risk of severe falls. This underscores the necessity for clinicians to carefully weigh the benefits and drawbacks of prescribing aspirin, particularly in elderly patients who may require long-term use [340].

Several clinically approved fenamate NSAIDs, such as flufenamic acid and mefenamic acid, have been shown to selectively inhibit the NLRP3 inflammasome. The underlying mechanism involves the inhibition of volume-regulated anion channels in macrophages, independent of COX enzymes. Research indicates that these drugs exhibit significant efficacy in mouse models of NLRP3-dependent inflammation, and they also demonstrate therapeutic potential in amyloid β-induced memory loss and transgenic AD mouse models [225]. In addition to their classical COX inhibitory effects, certain NSAIDs can activate PPARγ, inhibit NF-κB activation, or modulate protein response pathways, thereby further enhancing their anti-inflammatory effects [341].Notably, COX-1 inhibition in mice may help alleviate neuroinflammation and working memory impairment induced by β-amyloid [342], while the inducible expression of COX-2 plays a critical role in long-term synaptic plasticity [69, 257, 343].

Other drugs, such as aspirin and celecoxib, also show potential in neuroprotection and anti-inflammatory effects [240, 344, 345], although the role of aspirin in dementia and cognitive decline remains controversial [346]. Overall, fenamate NSAIDs and related compounds may represent a novel therapeutic direction for Alzheimer's disease, particularly through the inhibition of inflammasomes and neuroinflammation.

### 8.7 TNF-α Inhibitors

As shown in Table 3, Anti-TNF inhibitors (e.g., etanercept, adalimumab, infliximab) have been associated with a significantly reduced risk of AD in patients with RA [146, 347-349].

Three large-scale epidemiological studies have found that patients receiving etanercept treatment exhibit a 60% to 70% reduction in the incidence of AD. Two small RCTs indicate that etanercept can enhance cognitive performance in AD patients. A small RCT utilizing Theracurmin (a bioavailable form of curcumin) demonstrated improvements in cognitive performance, alongside reductions in levels of Aβ plaques and tau tangles. [148].

Research indicates that etanercept (ETN) enhances cell viability and neurite outgrowth while improving cognitive function in transgenic AD mouse models by inhibiting the JNK and NF-κB pathways [350]. Anti-TNF therapy has also been shown to improve BMD and microarchitecture in RA patients, reducing the risk of fractures [351]. These benefits may be modulated by serum 25-OH vitamin D and homocysteine levels [147].

Thirty-six patients with RA and seventeen patients with AS received treatment with ETN or certolizumab pegol (CZP) for a duration of one year. The inhibition of TNF-α was clinically effective, and anti-TNF-α therapy prevented further bone loss within the one-year period. Anti-TNF therapy significantly elevated levels of P1NP and SOST, while increasing the P1NP/βCTX ratio, and concurrently reduced the production of DKK-1 and CathK in the majority of patient cohorts. Anti-TNF treatment may exert opposing effects on DKK-1 and SOST. [145]. A novel biologic agent, TfRMAb-TNFR, which fuses the TNF-α receptor with transferrin receptor antibodies, demonstrated superior therapeutic effects compared to etanercept in transgenic APP/PS1 male mice at 10.7 months of age. TfRMAb-TNFR improved spatial reference memory and enhanced BBB tight junction protein expression. It effectively reduced insoluble Aβ deposition and microglial proliferation in the brain, even when administered as a delayed treatment. TfRMAb-TNFR achieved these outcomes without causing hematologic or iron homeostasis abnormalities, despite lower plasma exposure levels than etanercept [352]. Adalimumab has shown potential in reducing AD risk while treating RA and psoriasis. It has also demonstrated neuroprotective effects in a rat model of vascular dementia (VaD) by inhibiting neuroinflammation [348]. Similarly, infliximab alleviated AD-like pathology by modulating the TNF-α/Wnt/β-catenin signaling pathway and improved osteoporosis and tendon inflammation in RA patients [149, 349].

In summary, anti-TNF therapies not only help alleviate joint symptoms in RA patients but also hold promises for the prevention and treatment of AD. These findings underscore the potential of targeting TNF-α pathways in managing both inflammatory and neurodegenerative diseases.

### 8.8 Interleukin modulators

As shown in Table 3. Interleukin-1 receptor antagonist (IL1RN) is a critical immunomodulatory molecule that acts by competitively inhibiting the binding of IL-1 to its receptor IL-1R [353, 354]. Studies have demonstrated that IL1RN plays a regulatory role in osteoblasts by enhancing their differentiation and promoting the expression of osteogenic markers such as alkaline phosphatase, osterix, and osteocalcin [353]. Consequently, IL1RN has been identified as a novel therapeutic target for osteoporosis. IL1RN interacts with integrin β3, activating the β-catenin signaling pathway, which is instrumental in regulating osteoblast differentiation [355]. Beyond its implications in osteoporosis, IL1RN has shown promise in AD research. By antagonizing IL-1 receptors, IL1RN significantly improves cognitive function, reduces neurodegeneration associated with AD, and enhances synaptic function and memory performance in both healthy and AD model mice. Specifically, cognitive impairment induced by IL-1β in AD mouse models can be reversed by the IL-1 receptor antagonist anakinra [353]. Furthermore, anakinra has been found to mitigate mitochondrial dynamics alterations and synaptic loss induced by Aβ in mouse models [356].

IL-6 initiates cellular signaling through both membrane-bound and soluble receptor forms, contributing to inflammation and joint destruction in arthritis. IL-6 inhibitors, such as tocilizumab, have been validated as effective treatments for RA. These inhibitors significantly reduce joint inflammation and alleviate comorbidities such as osteoporosis, anemia, cardiovascular diseases, and depression [357]. By inhibiting IL-6 receptors, tocilizumab also improves tendon morphology and decreases inflammation-associated bone loss [358].

In summary, IL1RN and IL-6 inhibitors demonstrate substantial potential in treating a variety of immune-related diseases, particularly osteoporosis and Alzheimer's disease. These findings provide new perspectives and targets for future clinical therapies, underscoring their importance in addressing complex, multifactorial conditions [359].

### 8.9 CSF1R inhibitors

As shown in Table 3. Microglial cells play a pivotal role in CNS, functioning as both pro-inflammatory and anti-inflammatory mediators, and are therefore considered key regulators in central and peripheral neurodegenerative diseases [65]. Despite the morphological complexity of microglia, which makes their characterization challenging, they are promising therapeutic targets for neurodegenerative diseases [360]. As resident immune cells of the brain, microglia maintain neural homeostasis by monitoring their environment, and their activation is closely associated with pathological changes in the brain. CSF1R (colony-stimulating factor 1 receptor) signaling plays a critical role in intracellular amyloid accumulation and the formation of neuritic plaques—two events that are integral to the causal pathways of neurodegeneration and plaque formation [361]. CSF1R inhibitors, such as PLX3397, have demonstrated the ability to mitigate Aβ-induced impairments in long-term potentiation in AD mouse models. These inhibitors facilitate the transformation of microglia into a phagocytic phenotype, enhancing the clearance of Aβ and restoring neural transmission [362, 363]. PLX3397 has also been shown to significantly reduce microglial cell numbers and decrease Aβ plaque burden [364, 365]. Moreover, studies have indicated that high doses of another CSF1R inhibitor, PLX5622, can nearly eliminate microglial populations while improving learning and memory capabilities without causing cognitive deficits. This suggests that CSF1R inhibitors not only aid in removing dysfunctional microglia but also have a beneficial impact on cognitive function [366].

In summary, CSF1R inhibitors represent a promising therapeutic approach for treating neurodegenerative diseases. By targeting pathological microglial activity and facilitating Aβ clearance, these compounds hold potential for mitigating disease progression and enhancing cognitive outcomes in neurodegenerative conditions.

## 9. Conclusion

In this article, we elucidate the complex interplay between Alzheimer's disease (AD) and bone-related disorders, proposing the concept of an "inflammatory bridge" that connects neurodegenerative changes in the elderly with musculoskeletal pathology. Despite the traditionally limited clinical translational success associated with the focus on β-amyloid aggregation and tau protein hyperphosphorylation, recent evidence underscores the pivotal role of neuroinflammation, encompassing aberrant activation of microglia and astrocytes, disruption of the blood-brain barrier, and the involvement of systemic inflammatory mediators such as NLRP3 and CSF-1 in advancing AD pathology. Concurrently, persistent inflammation is linked to bone diseases, including osteoporosis, osteoarthritis, and rheumatoid arthritis. By emphasizing shared pathogenic pathways—specifically age-related NLRP3-mediated pyroptosis, advanced glycation end-products (AGEs), oxidative stress, macrophage dysregulation, and calcium ion imbalance—we propose that these converging processes form an "inflammatory bridge" that integrates cerebral and skeletal pathology. This perspective not only reveals common mechanisms but also identifies potential novel therapeutic intervention targets, including emerging anti-inflammatory agents and biomarkers, as well as the latest clinical trials of drugs that may simultaneously address neurodegenerative diseases and bone inflammatory disorders.
